# Triptycene-terminated thiolate and selenolate monolayers on Au(111)

**DOI:** 10.3762/bjnano.8.91

**Published:** 2017-04-20

**Authors:** Jinxuan Liu, Martin Kind, Björn Schüpbach, Daniel Käfer, Stefanie Winkler, Wenhua Zhang, Andreas Terfort, Christof Wöll

**Affiliations:** 1 Institute of Artificial Photosynthesis, State Key Laboratory of Fine Chemicals, Dalian University of Technology, 116024 Dalian, China; 2 Institut für Anorganische und Analytische Chemie, Goethe-Universität Frankfurt am Main, 60325 Frankfurt, Germany; 3 Physikalische Chemie I, Ruhr-Universität Bochum, 44780 Bochum, Germany; 4 National Synchrotron Radiation Laboratory, University of Science and Technology of China, Hefei, Anhui 230029, China; 5 Institute of Functional Interfaces, Karlsruhe Institute of Technology (KIT), 76021 Karlsruhe, Germany

**Keywords:** organic thin films, selenolates, self-assembled monolayers, thiolates, triptycene

## Abstract

To study the implications of highly space-demanding organic moieties on the properties of self-assembled monolayers (SAMs), triptycyl thiolates and selenolates with and without methylene spacers on Au(111) surfaces were comprehensively studied using ultra-high vacuum infrared reflection absorption spectroscopy, X-ray photoelectron spectroscopy, near-edge X-ray absorption fine structure spectroscopy and thermal desorption spectroscopy. Due to packing effects, the molecules in all monolayers are substantially tilted. In the presence of a methylene spacer the tilt is slightly less pronounced. The selenolate monolayers exhibit smaller defect densities and therefore are more densely packed than their thiolate analogues. The Se–Au binding energy in the investigated SAMs was found to be higher than the S–Au binding energy.

## Introduction

**Figure 1:**
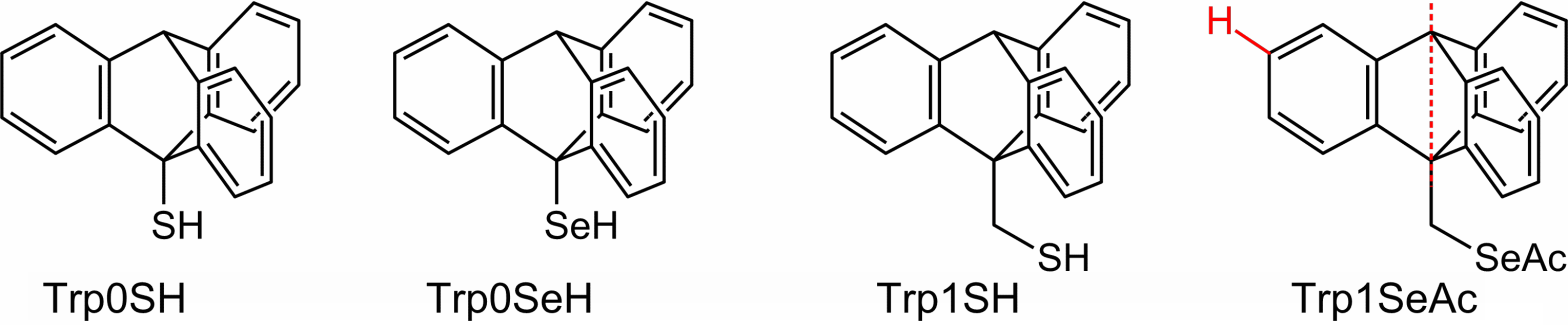
Schematic representation of the triptycene-based sulfur and selenium compounds used for the formation of SAMs on Au(111) surface. Exemplary, in the Trp1SeAc structure, the main molecular axis is shown as a dotted red line. In this structure, also the H atom bound to the C^3^ atom is shown. This H atom has the highest distance to the chalcogen anchor group, which defines the maximum possible monolayer thickness. Both molecular axis and theoretical maximum monolayer thickness are used in the discussion.

For more than three decades now, self-assembled monolayers (SAMs) have enjoyed ever-increasing popularity in the modification and functionalization of surfaces. Numerous applications of SAMs have been reported, e.g., in electrochemistry [1–2], molecular electronics [1–2] and biocompatibility [1–2], to name only a few. Particularly interesting is the use of SAMs as templates for crystallographically oriented metal–organic framework (MOF) thin film growth [3]. The by far most intensely studied SAM systems are those of organothiolates on gold surfaces [1,4]. More recently, selenolate SAMs on gold have experienced considerable attention because of their superior degree of order in comparison to their thiolate analogues [5–8]. The structure and degree of order in SAMs strongly depend on the nature of the backbones of the SAM-building molecules [1,4]. It is well-known that long-chained alkyl moieties [9] promote the formation of well-ordered thiolate monolayers on gold via van der Waals forces. Another example for highly crystalline monolayers are biphenyl [10–12] and terphenyl [13–14] thiolates on gold, which exhibit strong π-stacking. More bulky moieties like adamantyl [15–17] show noteworthy less attractive intermolecular interactions, which is rather disadvantageous for the formation of well-ordered monolayers. Furthermore, the increased space requirement of the adamantyl moiety only permits relatively low packing densities of thiolates or selenolates, making SAMs of such molecules energetically unfavorable. Consequently, less bulky thiolates/selenolates are able to replace adamantyl-terminated SAMs on gold [18–19]. Thus, the preparation of thiolate and selenolate monolayers with voluminous organic moieties represents a certain challenge. Still, such SAMs are worthwhile to be investigated from a fundamental point of view. Moreover, the use of bulky SAMs allows for tuning the surface density of functional groups, like –COOH, which is meaningful, e.g., in the context of surface-mounted MOFs (SURMOFs). Recently, it has been shown that the growth of HKUST-1 on –COOH-terminated triptycene (Trp)-based SAMs proceeds along a different crystallographic axis than the growth on SAMs with a higher surface density of –COOH-functions, indicating that the surface density of –COOH units is a factor to liquid phase epitaxy of SURMOFs [20]. The temperature has another crucial impact on the efficiency and the direction of MOF growth on surfaces [3,21]. The strength of the chalcogenate–gold bond represents an upper limit to the MOF deposition temperature. Thus, replacing thiolate by selenolate with its stronger bond to gold [7,22–25] might open an opportunity to increase the temperature range for liquid epitaxy of MOFs. The insertion of a methylene spacer group between the anchor group and aromatic moieties has been reported to improve the structural quality of, e.g., anthracene-based SAMs [26–27]. For this reason, we aimed to systematically investigate how the structure, stability and the order of monolayers of molecules with the highly space-demanding triptycene moiety depend on the anchor group (thiolate vs selenolate) and how they are influenced by the presence or absence of a spacer group, i.e., a methylene unit, between the chalcogen atom and the Trp unit. To this end, the molecules displayed in Figure 1, 9-triptycenethiol (Trp0SH), 9-triptyceneselenol (Trp0SeH), (9-triptycene)methylthiol (Trp1SH) and (9-triptycene)methylselenoacetate (Trp1SeAc) were deposited out of ethanolic solution onto Au(111) surfaces. The resulting films were investigated using a complementary set of spectroscopic methods, namely ultra-high vacuum (UHV) infrared reflection–absorption spectroscopy (IRRAS), X-ray photoelectron spectroscopy (XPS), near-edge X-ray absorption fine structure (NEXAFS) spectroscopy and thermal desorption spectroscopy (TDS).

## Results

### Synthesis of Trp1SeAc

While the syntheses of the other three target molecules have been described in scientific journals [28–29], Trp1SeAc has until now only been reported in the PhD thesis of one of us (B. S.) [30]. For this reason, we give some detailed information on its synthesis.

Owing to the air sensitivity of aliphatic selenols, we did not try to synthesize the selenol Trp1SeH but aimed for a related, protected compound. Since the diselenide (Trp1Se)_2_ is rather insoluble, we decided to turn it into the selenoacetate Trp1SeAc (compare Scheme S1 in the Supporting Information File 1). Selenoacetate compounds can be used to form selenolate SAMs on gold surfaces [8]. Earlier work on homologous sulfur compounds revealed that free thiol groups are incompatible with benzyne [28]. Yet, it could be shown that formation of the triptycene group by reaction of anthracene moieties with benzyne is possible if the sulfur atom is protected, e.g., with an acyl group [28]. We suspected the selenol group to be similarly unstable towards benzyne as the thiol group and thus aimed to protect it. For this purpose, 9-(chloromethyl)anthracene was reacted with potassium selenocyanate to yield 9-anthrylmethylselenocyanate. From the reaction of this substance with benzyne, 9-triptycylmethylselenocyanate could be obtained in a reasonable yield (see Supporting Information File 1). The cleavage of the protecting cyanide group could be achieved by reacting 9-triptycylmethylselenocyanate with sodium borohydride and subsequent oxidation of the product under ambient conditions (compare Supporting Information File 1). The diselenide obtained this way was reduced with elemental sodium using benzophenone as a catalyst, and reacted with acetyl chloride to yield the selenoacetate Trp1SeAc.

### IR Spectroscopy

**Figure 2:**
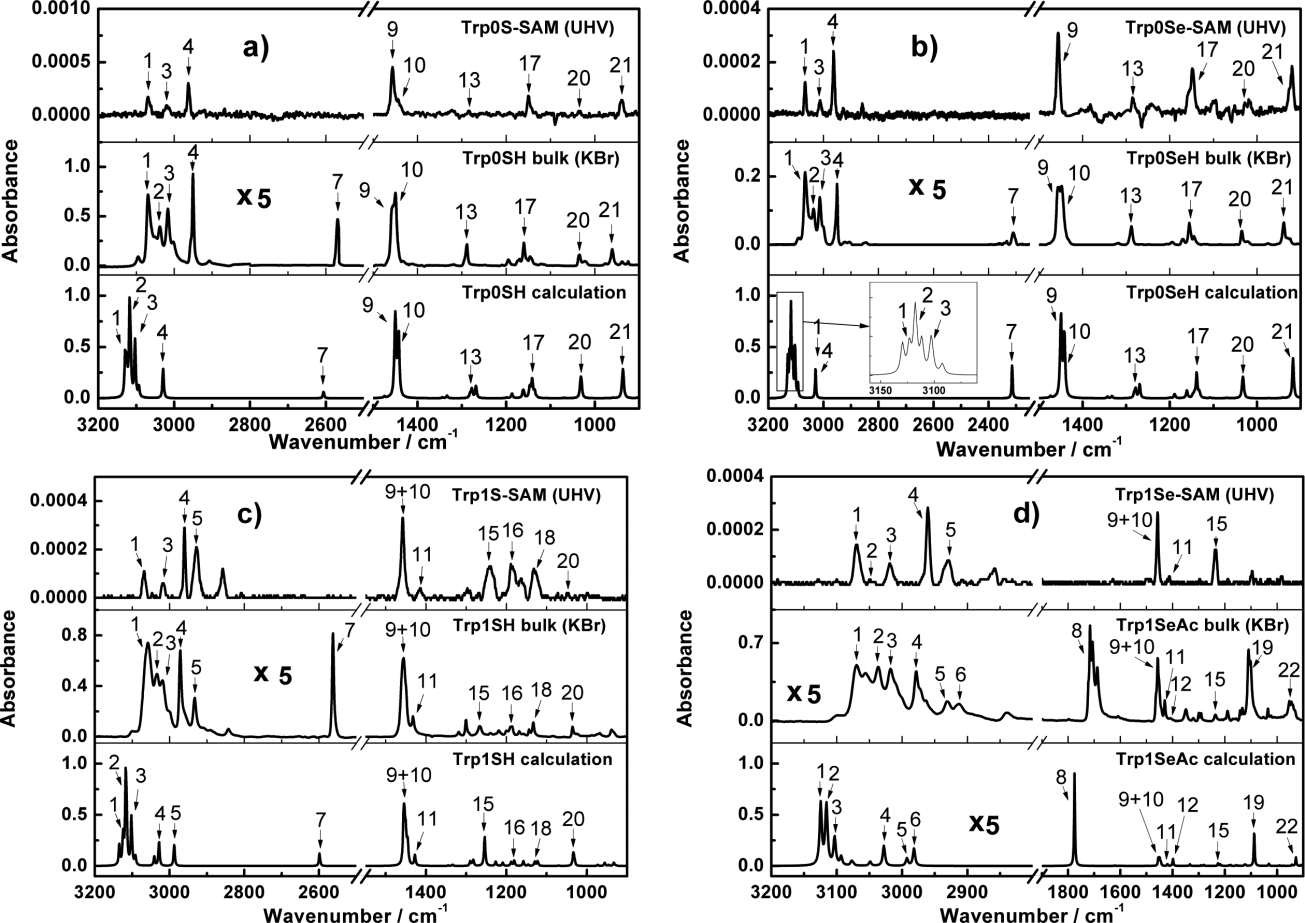
Experimental and calculated spectra of (a) Trp0S-, (b) Trp0Se-, (c) Trp1S-, and (d) Trp1Se-species. In all panels, the upper rows contain the UHV-IRRA spectra of the respective thiolate/selenolate-SAMs on Au(111), the middle rows the KBr pellet spectra and the lower rows the calculated spectra of the isolated molecules displayed in Figure 1. The calculated spectra are given in arbitrary units of absorption.

In Figure 2, IRRA spectra recorded from Trp0S-, Trp0Se-, Trp1S- and Trp1Se- layers on gold substrates (upper panel) as well as IR spectra from KBr pellets (middle panel) and calculated spectra of the corresponding molecules (lower panel) are shown. The most intense bands in these spectra are numbered and their wavenumber positions and assignments are listed in Table 1.

**Table 1:** Assignment of some bands obtained from the calculated, KBr pellet and SAM IR spectra of triptycene-terminated species along with the orientation of their transition dipole moments (TDMs) relative to the main molecular axis as deduced from the calculations.^a^

Band position / cm^−1^														
No	Assignment^b^	TDM^c^	Trp0S			Trp0Se			Trp1S			Trp1Se		
			Calc.	KBr	SAM	Calc.	KBr	SAM	Calc.	KBr	SAM	Calc.	KBr	SAM

1	ν CH arom	_|_	3129	3068	3069	3123	3066	3066	3127	3058	3069	3123	3069	3070
4	ν CH aliph	||	3030	2950	2962	3026	2950	2963	3029	2972	2960	3028	2978	2961
5	ν CH_2_ as	_|_	–	–	–	–	–	–	2988	2933	2926	2992	2931	2930
7	ν SH/ν SeH		2606	2569	–	2315	2310	–	2598	2562	–	–	–	–
8	ν CO		–	–	–	–	–	–	–	–	–	1774	1710	–
10	δ CH arom	_|_	1442	1450	1444	1442	1448	1442^d^	–	–	–	–	–	–
13	δ CH arom	_|_	1276	1287	1285	1278	1287	1286	1290	1319	1322	–	–	–
14	δ CH arom	||	1268	1287	1285	1269	1287	1288	1281	1301	1296	–	–	–
16	ν CC	||	–	–	–	–	–	–	1180	1189	1186	–	–	–
17	δ CH ν CC arom	_|_	1161	1169	1150	1160	1156	1157	–	–	–	–	–	–
18	δ CH arom	||	1141	1146	1135	1139	1145	1148	1129	1133	1130	1123	1134	–
20	ν CC ρ arom	||	1032	1035	1036	1032	1035	1027	1033	1037	1049	1033	1036	1034
21	ν CS/ν CSe	||	937	961	940	917	939	920	–	–	–	–	–	–

^a^Note that this table is an excerpt of Table S1 in the Supporting Information File 1, that contains assignments of an extended number of bands. ^b^ν: stretching, δ: bending, γ: wagging, ρ: rocking; arom: aromatic, aliph: aliphatic, s: symmetric, as: asymmetric. ^c^_|_: perpendicular or almost perpendicular to main molecular axis, ||: parallel or almost parallel to main molecular axis, /: neither parallel nor perpendicular to main molecular axis. ^d^Shoulder.

The calculated spectra can be used to assign the experimentally found bands to vibrational modes. All calculated spectra are in good agreement with the corresponding KBr pellet spectra, in particular in the region below 1500 cm^−1^. The IRRA spectra of the molecular layers feature a lot of the bands that can be found in the calculated and in the KBr pellet spectra, indicating the presence of the triptycene-based target molecules on the gold surface. The bands 7 at 2569 cm^−1^ (Trp0SH), 2562 cm^−1^ (Trp1SH) and 2310 cm^−1^ (Trp0SeH) associated with the respective S–H and Se–H stretching modes and band 8 at 1710 cm^–1^ assigned to the C=O stretching mode due to the acetyl protecting group (Trp1SeAc) are completely absent in the spectra of the SAMs, by this indicating S–H, Se–H and Se–Ac bond cleavage and formation of thiolates and selenolates on the gold surface upon immersion of the substrates into the solutions of the triptycene-terminated molecules. This finding confirms the formation of triptycene-based molecular layers in case of all investigated molecules.

IRRAS can often be used not only to identify the nature of molecular species adsorbed on surfaces but also to gain information on molecular alignment with respect to the surface normal. Due to screening effects, only the transition dipole moment (TDM) vector component perpendicular to the substrate surface contributes to the IRRAS signal intensity of the vibrational bands. This phenomenon is generally referred to as surface selection rule on metals [31]. As a consequence of this rule, bands with TDMs oriented perpendicular to the substrate surface have high relative intensities in the IRRA spectra, while bands with TDMs (mostly) parallel to the surface will be attenuated or even completely extinguished. Thus, from comparison of relative band intensities in neat substance spectra to those in spectra of monolayers, information can be gained on the alignment of the molecules. With the help of DFT calculations, in the IR spectra of the triptycene-based molecules, two types of vibrational bands can be identified. The TDMs of the first type (designated || in Table 1) are parallel to the molecular main axis that passes through both aliphatic C atoms of the triptycene unit (compare Figure 1). The other type of transitions exhibits TDMs perpendicular to this axis (designated _|_ in Table 1). The angle of the main molecular axis and the substrate surface normal is referred to as the tilt angle β.

In the IRRA spectra of the triptycene-based layers, neither the || bands (4, 14, 16, 18, 20, 21, see Table 1) nor the _|_ bands (1, 5, 10, 13, 17, see Table 1) show a marked, systematic attenuation. From this, it can be inferred that the molecules are not upright (β = 0°) but significantly tilted.

### XPS

**Figure 3:**
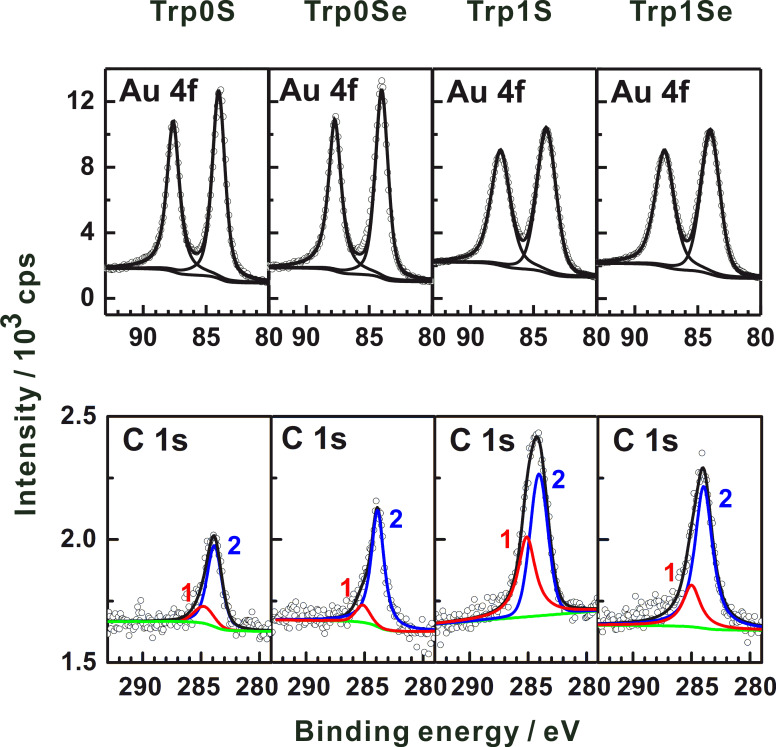
XP spectra of triptycene-terminated thiolate- and selenolate-monolayers on Au(111). See text for discussion.

XP spectra recorded from the Trp0S-, Trp0Se-, Trp1S- and Trp1Se molecular layers on gold are displayed in Figure 3. Unfortunately, due to the limitations of our XPS apparatuses, we were not able to obtain the S 2p and Se 3p signals of the investigated triptycene-based layers with a quality allowing for a quantitative determination of coverages. Note that the *S*/*N* ratio can in principle be improved by recording a greater number of spectra. However, it is well-known that organic monolayers can be damaged by exposure of X-ray photons for such long measuring times (cleavage of C–S bonds, see [2]). Although the presence of S and Se in the thiolate and selenolate layers, respectively, is evidenced by thermal desorption spectroscopy (see below), an evaluation of the S and Se XP signals might have been useful for determination of packing densities. However, a direct comparison of the S to Se signals might be spoiled by photoelectron diffraction effects [32] of different extent. However,with the available instrumentation, C 1s signals could be detected in a straightforward fashion. The obtained C 1s binding energies of about 284 to 285 eV (Table 2, see below) are typical of carbon atoms in aliphatic and aromatic compounds, i.e., the XPS data are in line with the IR data, confirming the formation of triptycene-based layers on the Au(111) surface. The low quality of the XP spectra made us refrain from a detailed (e.g., fitting of different components) analysis of the C 1s signals. Still, these data can be used to obtain layer thicknesses. XPS is a widely used method to determine layer thicknesses of SAMs [2]. Other methods with sufficient height resolutions (0.1 nm) are ellipsometry and scanning probe microscopy (SPM). To our knowledge, SPM methods have so far not been successfully used to determine the absolute thickness of organic self-assmbled monolayers, most likely due to difficulties with preparing a defined border between zones with and without the molecules on the substrate. Occasionally, using sophisticated patterning methods, height differences between two different SAMs have been determined [33]. Ellipsometry is suitable to measure thicknesses of monomolecular films, in principle, but the determination of crucial parameters like the complex refractive index of the SAM-forming molecule is not straightforward. The small amounts of the synthesized triptycene-based molecules did not allow for obtaining these parameters, thus making the use of estimated values necessary. In light of these considerations, XPS is the most appropriate method to determine the thicknesses of the investigated layers.

Thicknesses of the Trp-based molecular layers could be obtained by comparing the ratios of the Au 4f and the C 1s intensities (*I*_Au_ and *I*_C_) in the spectra of the investigated Trp-based layers with the respectice ratio in a spectrum of a reference with known thickness [34]. An *n*-decanethiolate SAM on Au with a thickness of 13.1 Å [9] served as the reference. To obtain *I*_C_ in the spectra of the Trp-layers two Gaussian functions (labeled 1 and 2 in Figure 3) were fitted to the C 1s signals using a Shirley background (green traces in Figure 3) in the XP spectra of all four triptycene-based molecular layers. Note that the purpose of these Gaussian functions is restricted to the evaluation of the overall C 1s intensities. For the reason mentioned above, no attempt was made to draw conclusions from the energetic positions of their maxima or from their individual intensities. The thicknesses of the molecular films were deduced by applying Equation 1 [34].

[1]
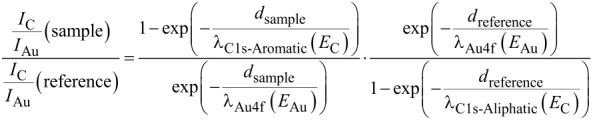


Here, the photoelectron escape depths λ of gold and carbon depend on the X-ray source, for Al Kα (1486.6 eV), they amount to λ_Au4f_ = 45 Å at a photoelectron kinetic energy of *E*_Au_ = 1402 eV [35] and λ_C1s-Aliphatic_ = 35 Å [36], λ_C1s-Aromatic_ = 27.3 Å [37] at a photoelectron kinetic energy of *E*_C_ = 1202 eV. Note that the smaller escape depth in case of aromatic layers arises from a higher electron density compared to aliphatic layers. The value of 27.3 Å results from the comparison of XP spectra of SAMs with known thicknesses (an *n*-alkanethiolate monolayer and a terphenylthiolate monolayer) [37].

**Figure 4:**
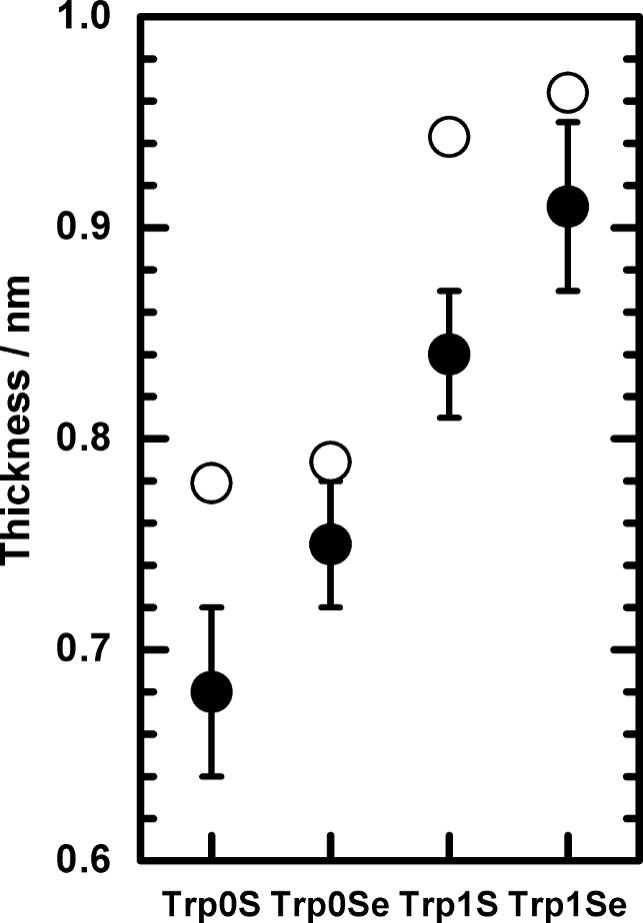
Thicknesses of the Trp0S, Trp0Se, Trp1S and Trp1Se monolayers on Au(111) determined from the evaluation of the XPS data (●) and theoretical maximum monolayer thicknesses (○) (see text).

The resulting experimental thicknesses of all triptycene-based films are listed in Table 2, along with theoretical maximum monolayer thicknesses. The latter ones are thought to be identical to the highest distance that could be found between the chalcogen atom and any other atom in the respective molecules, plus the respective covalent radii of the two atoms. These distances were obtained from molecular structures generated by applying standard bond lengths and angles. The H atom connected to the C^3^ atom (compare Figure 1) was found to possess the largest distance to the chalcogen atom in case of all four molecules. The experimental thicknesses and the maximum monolayer thicknesses assessed by this way are displayed in Figure 4. For all target molecules, the layer thicknesses are only slightly smaller than the assumed maximum for monolayers, which points to monolayer formation on the Au(111) surface. Notably, the difference between the measured and the theoretical maximum monolayer thicknesses is more pronounced in case of the S-anchored Trp0S and Trp1S layers as compared to the Se-anchored TrpSe0 and TrpSe1 films.

**Table 2:** Results of the evaluation of XP spectra recorded from Trp0S-, Trp0Se-, Trp1S- and Trp1Se-SAMs on Au(111). The full width at half maximum values for XPS data fitting are given in braces. Additionally to the experimental layer thicknesses that result from the XP data, a theoretically possible maximum monolayer thickness is given. See text for explanation.

Sample	Binding energy / eV			Intensity / cps			Thickness / Å	
	Au 4f_7/2_	C 1s (1)	C 1s (2)	Au 4f_7/2_	C 1s (1)	C 1s (2)	experimental	max.^a^

Trp0S	84.0 (1.2)	283.9 (1.3)	285.1 (1.3)	18173	750	199	6.8 ± 0.4	7.79
Trp0Se	84.0 (1.2)	283.9 (1.3)	285.1 (1.3)	17651	856	184	7.5 ± 0.3	7.89
Trp1S	84.0 (1.8)	284.1 (1.8)	285.1 (1.8)	37326	1189	919	8.4 ± 0.3	9.43
Trp1Se	84.0 (1.8)	284.0 (1.8)	285.0 (1.8)	37247	1531	480	9.1 ± 0.4	9.64

^a^Theoretical maximum monolayer thickness, based on the respective molecular dimensions as obtained from application of standard bond lengths and angles.

### NEXAFS spectroscopy

**Figure 5:**
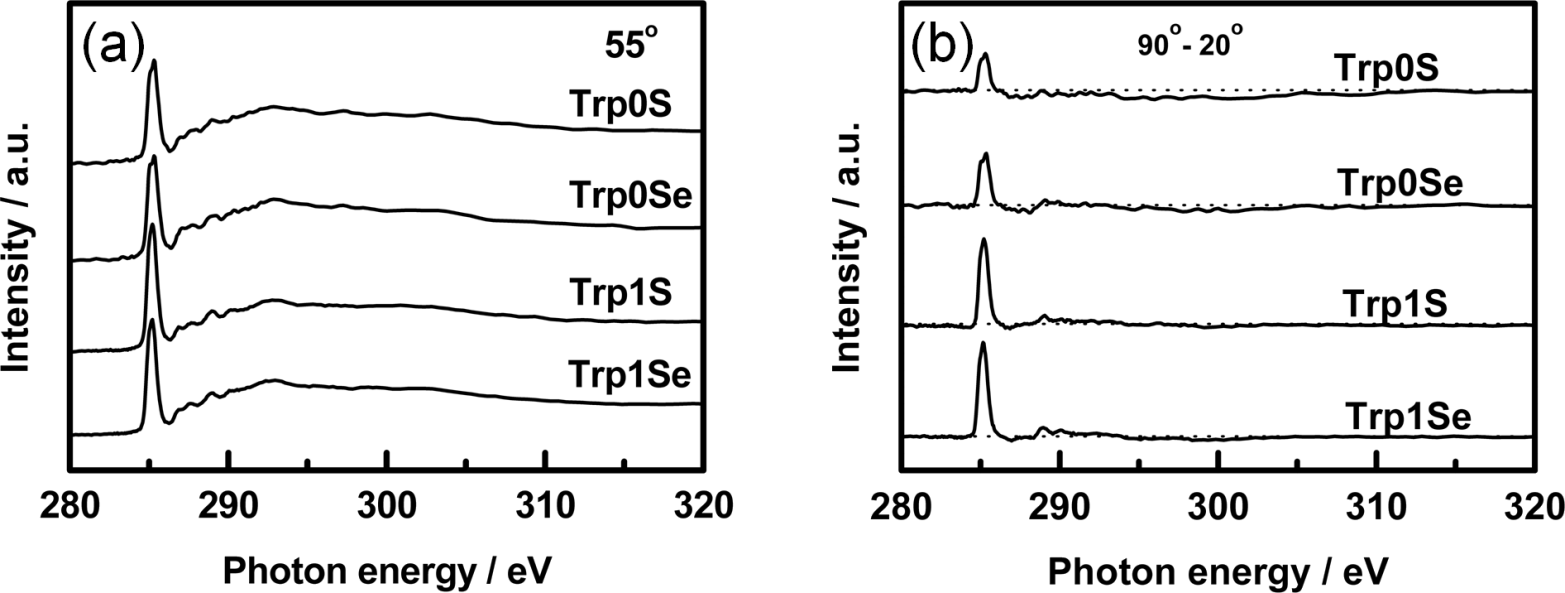
Carbon K-edge NEXAFS spectra of Trp0S-, Trp0Se-, Trp1S- and Trp1Se-SAMs on Au(111). (a) Recorded at an X-ray incidence angle of 55°. (b) Difference between the spectra recorded at X-ray incidence angles of 90° and 20°. The horizontal dotted lines correspond to zero.

Carbon K-edge NEXAFS spectra of the Trp0S-, Trp0Se-, Trp1S-, and Trp1Se-SAMs were recorded at different incidence angles of the X-ray beam and are displayed in Figure 5.

For all four monolayers, a pronounced dichroism is evident, i.e., the intensity of single resonances is strongly dependent on the X-ray incidence angle θ. This points to a uniform alignment of the thiolate and selenolate molecules on the Au(111) surface. A couple of absorption resonances are visible in the NEXAFS spectra, the strongest of which is the C 1s-π_1_* resonance in the aromatic rings, located around 285 eV. Other, less prominent maxima at higher energies (287.0 eV and 287.8 eV, etc.) can be assigned to the excitation of C 1s electrons into other π* orbitals or Rydberg states [38]. In the present study, we focus on the C 1s-π_1_* transition since it has the most intense signals and it is highly dependent on the incidence angle of X-ray irradiation and thus allows for evaluation of average tilt angles β of the molecules in the SAMs.

**Figure 6:**
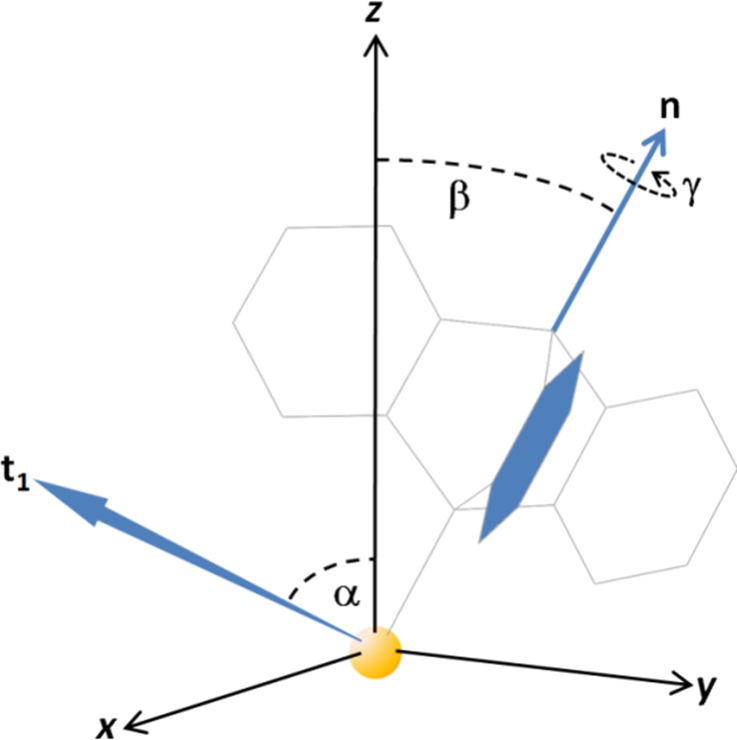
Illustration of relevant angles in a triptycene-based molecule anchored on a gold surface. The chalcogen atom is located in the origin of the coordinate system, the *z*-axis represents the substrate surface normal. The main axis **n** of the molecule is tilted against the surface normal (identical to the *z*-axis) by the angle β. The transition dipole moment **t_1_** related to an example aromatic ring (highlighted in blue) is tilted against the surface normal by the angle α. The rotation of the molecule about the main axis is given by γ. Further details can be found in the Supporting Information File 1.

Usually, the value of β cannot be directly obtained from evaluation of NEXAFS spectra. Instead, with NEXAFS, the spatial orientation of the C 1s-π_1_* transition dipole moment can be determined, namely the angle α between the TDM and the substrate surface normal. For SAMs with the main molecular axis perpendicular to the C 1s-π* TDM, the angles α and β are related by [39–40]:

[2]
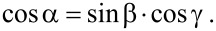


Here, γ is the twist angle of the aromatic ring with respect to the plane spanned by the surface normal and the molecular axis, defined such that γ = 0 if the TDM lies in the aforementioned plane. This third parameter, γ, is an obstacle to the determination of molecular tilt since in general it demands additional information (either geometric assumptions or input from complementary experimental methods or theoretical calculations). Only in rare cases, β can unambiguously be determined from NEXAFS data, e.g., by evaluation of two transitions with perpendicular TDMs [8,40]. Sometimes, the combination of IRRAS, NEXAFS experiments and theoretical calculations of NEXAFS spectra allows for reliable conclusions on the molecular tilt in SAMs [41]. In the present study, the high (3-fold) symmetry of the triptycene moiety permits the unambiguous determination of β, yet renders any information on γ inaccessible: Assuming that the three aromatic rings of the triptycene unit produce independent NEXAFS resonance signals of equal intensity with TDMs rotated against each other by 120° steps, the X-ray incidence angle θ and the tilt angle β are related by:

[3]
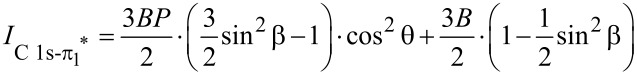


Here, *B* is a scaling factor, and *P* is the degree of polarization of the synchrotron X-ray radiation. Based on Equation 3, β can be determined by plotting the relative NEXAFS intensities against cos^2^θ. Straight lines can be fitted to the data, from the slopes *m* and intercepts *a* of which, β can be calculated using:

[4]
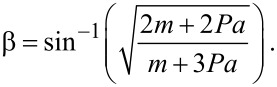


For derivation of Equation 3 and Equation 4, see the Supporting Information File 1.

**Figure 7:**
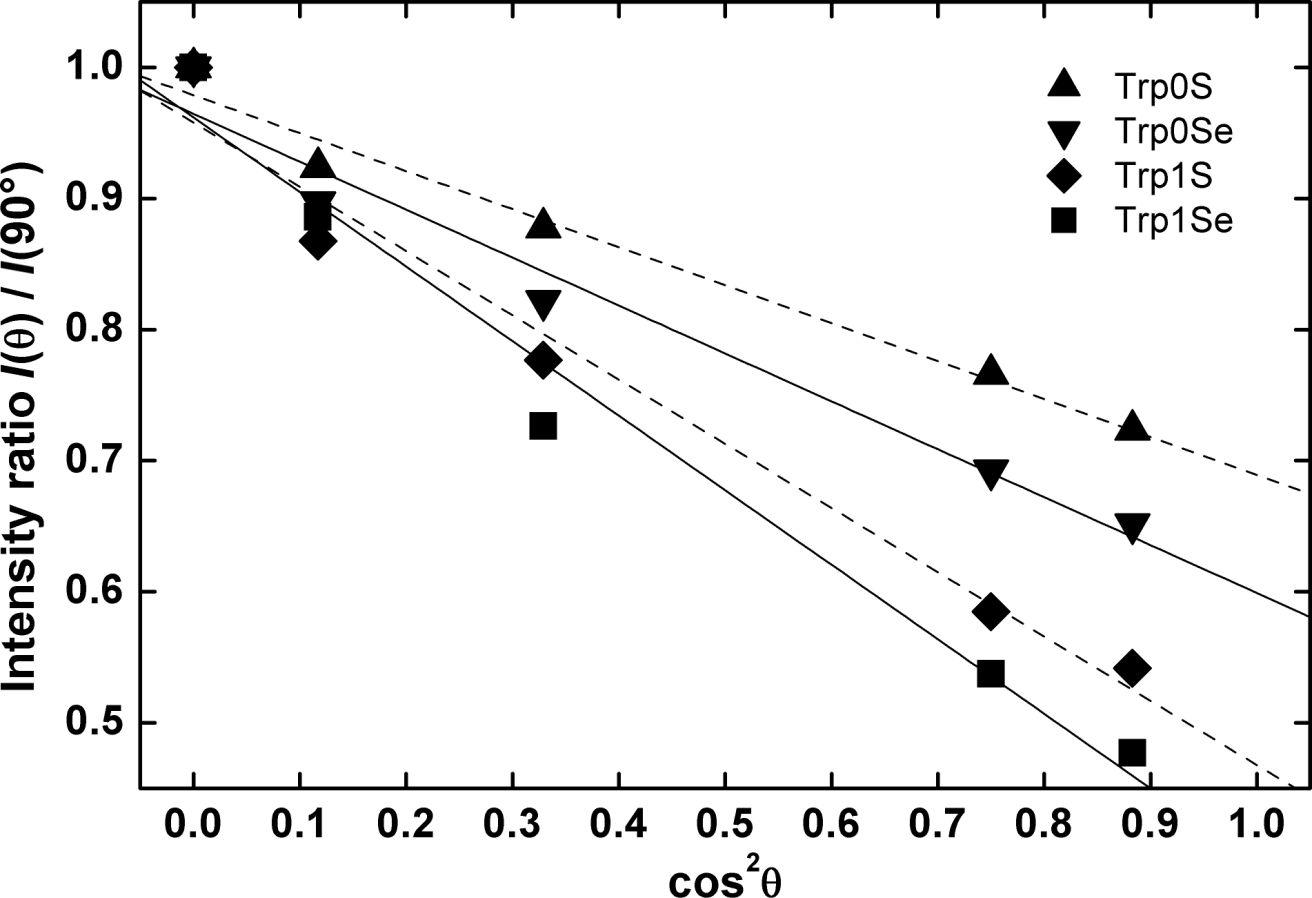
NEXAFS C 1s-π^*^ resonance intensity ratio *I*(θ)/*I*(90°) of Trp0S- (▲), Trp0Se- (▼), Trp1S- (♦) and Trp1Se- (■) SAMs on Au(111) dependencies on the square of the cosine of the incidence angle θ along with the least-squares fitting curves. The molecular tilt angles relative to the substrate surface normal as obtained by the fitting of the NEXAFS intensities are listed in Table 3.

In Figure 7, the NEXAFS C 1s-π_1_* resonance intensity ratios *I*(θ)/*I*(90°) of the triptycene-based SAMs are plotted against cos^2^θ. The straight lines (also in Figure 7) obtained from fitting the data points can be evaluated to yield values for β as listed in Table 3. As already inferred from the IR data, the molecules in the four monolayers are substantially tilted with the Trp0S-SAM having the highest tilt angle of β = 44° and the Trp1Se-SAMs the lowest one with β = 33°.

**Table 3:** Tilt angles β of the molecular axes of the triptycene units obtained from the C 1s-π_1_* resonance in the NEXAFS spectra of Trp0S, Trp0Se, Trp1S and Trp1Se-SAMs on Au(111). Errors are given on 2σ level.

SAM	Trp0S	Trp0Se	Trp1S	Trp1Se

β / °	44 ± 4	41 ± 6	37 ± 6	33 ± 7

### TDS

**Figure 8:**
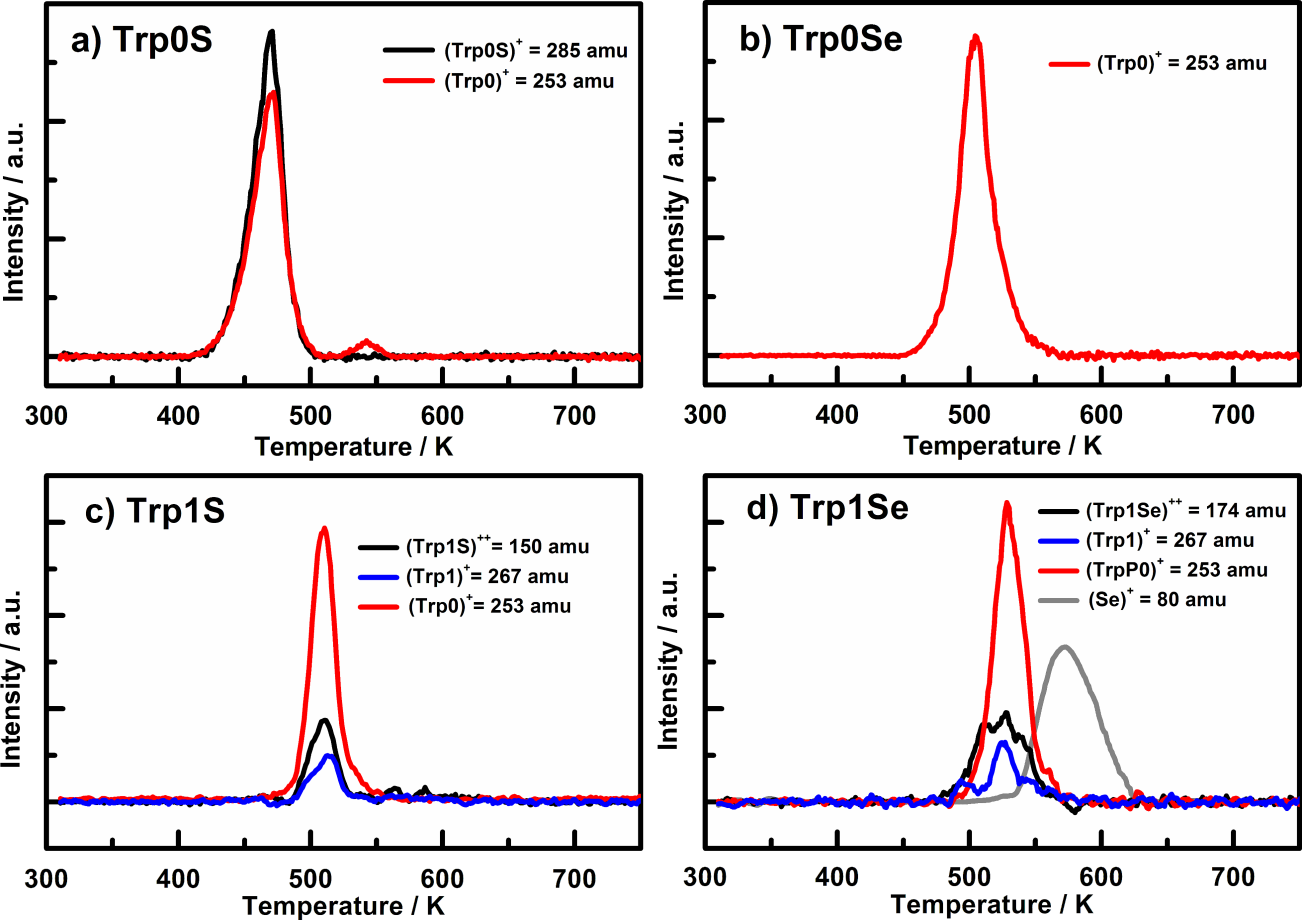
Thermal desorption spectra of a) Trp0S-, b) Trp0Se-, c) Trp1S- and d) Trp1Se-SAMs on Au(111). See text for discussion.

TDS was performed to obtain desorption paths and energies of the investigated triptycene-terminated monolayers. Data were acquired at the masses of S^+^ (*m*/*z* = 32), Se^+^ (*m*/*z* = 79), triptycyl^+^ (*m*/*z* = 253), triptycylmethyl^+^ (*m*/*z* = 267) and triptycylsulfur^+^ (*m*/*z* = 285). Since the molecular masses of the other sulfur- and selenium-based fragments are close to or even beyond the maximum detectable mass of the used apparatus, we scanned the molecular masses divided by two to detect the doubly charged molecular fragments: *m*/*z* = 150 (triptycylmethylsulfur^++^), *m*/*z* = 166 (triptycylselenium^++^) and *m*/*z* = 173 (triptycylmethylselenium^++^). The resulting spectra are displayed in Figure 8.

The TD spectrum of the Trp0S-SAM (Figure 8a) exhibits an intense signal at *m*/*z* = 285 with a peak temperature of 469 K, indicating the desorption of the intact molecule. At the same temperature, also an *m*/*z* = 253 signal could be detected. The equality in temperature suggests that already desorbed Trp0S molecules undergo fragmentation during the detection process in the mass spectrometer. Another, much less intense signal at *m*/*z* = 253 and 542 K is likely due to desorption of triptycyl fragments from defects. No unambiguous indication of atomic sulfur desorption could be found, probably because of interference with residual O_2_ in the UHV chamber. In the TD spectrum of the Trp0Se-SAM (Figure 8b), a strong *m*/*z* = 253 signal at 504 K was detected, and no *m*/*z* = 166 (triptycylselenium^++^) signal whatsoever. This finding points to desorption under cleavage of the Se–C bond. However, remaining selenium atoms could not be clearly detected, only a broad, very weak signal at *m*/*z* = 79 (probably from Se) was found in the region 500–625 K (not shown in Figure 8b). The TD spectrum of the Trp1S SAM (Figure 8c) shows signals at *m*/*z* = 253 (triptycyl^+^), 267 (triptycylmethyl^+^) and 150 (triptycylmethylsulfur^++^) with almost identical maximum temperatures (509 K, 512 K, 510 K). It remains unclear if different desorption mechanisms occur or if desorbed Trp1S molecules decompose during the detection process. However, the similarity of the maximum temperatures rather suggests the latter. As in the case of the Trp0S-SAM, no clear indication of atomic sulfur desorption could be found. The TD spectrum of the Trp1Se SAM (Figure 8d) is quite similar to the one of Trp1S yet with slightly higher desorption temperatures (529 K at *m*/*z* = 253 (triptycyl^+^), 527 K at *m*/*z* = 267 (triptycylmethyl^+^) and 530 K at *m*/*z* = 173 (triptycylmethylselenium^++^), respectively). An additional, quite strong and rather broad signal at *m*/*z* = 79 with a peak temperature of 575 K is a clear indication that atomic selenium remains on the surface upon temperature-induced desorption of the organic moiety of the Trp1Se SAM at ≈530 K and comes off at higher temperatures.

From the TDS data, activation energies of desorption *E*_des_ can be obtained using the Redhead formula:

[5]
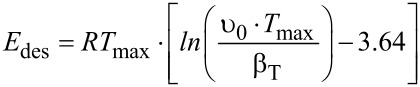


with *T*_max_ = the maximum of the TDS signal at a given mass and β_Τ_ = the applied heating ramp. The pre-exponential factor ν_0_ can be determined experimentally by variation of the heating rate (using the Polanyi–Wigner equation), requiring a variation by more than one order of magnitude for accurate results, which is a rather challenging task. However, it could be shown that ν_0_ can be estimated fairly well by using the integrated Clausius–Clapeyron equation ν_0_ = *p*_0_/σ(2π*mkT*)^1/2^ [42] and tabulated values for the equilibrium vapor pressure *p*_0_ taken from Antoine fit parameters of the vapor pressure [43–45]. The latter procedure yields values of 8.0 × 10^16^ s^−1^ for the Trp0 species and 8.8 × 10^16^ s^−1^ for the Trp1 species, which have been used as best estimations in further calculations of activation energies of desorption of the triptycene-based molecules. The desorption energies obtained by using Equation 5 are listed in Table 4, together with the maximum desorption temperatures.

**Table 4:** Experimental desorption temperatures and energies as derived from the TDS data of the Trp0S-, Trp0Se-, Trp1S- and Trp1Se-SAMs.

*m*/*z*	fragment	*T*_max_ / K				*E*_des_ / eV			
		Trp0S	Trp0Se	Trp1S	Trp1Se	Trp0S	Trp0Se	Trp1S	Trp1Se

173	(Trp1Se)^++^	–	–	–	523	–	–	–	1.91
166	(Trp0Se)^++^	–	–	–	–	–	–	–	–
150	(Trp1S)^++^	–	–	509	–	–	–	1.86	–
285	(Trp0S)^+^	469	–	–	–	1.70	–	–	–
267	(Trp1)^+^	–	–	512	527	–	–	1.86	1.92
253	(Trp0)^+^	469 542	504	510	530	1.70 1.97	1.83	1.86	1.93
79	Se^+^	–	–	–	575	–	–	–	2.10

## Discussion

The objective of the present work is the analysis of the structure and the energetical properties of the investigated triptycene-based layers on the Au(111) surface, and how they depend on the nature of the anchor group and the presence of a methylene spacer group in the molecules. It is obvious that in all four cases ordered monolayers are formed: The presence of the triptycene-based molecules on the surface is demonstrated by their vibrational signatures in the IRRA spectra (Figure 2). IRRAS also gives evidence of the binding mode of the molecules to the gold surface, namely formation of the thiolates and selenolates from the respective thiols or selenols. This is in complete analogy to numerous other sulfur-based [1] and selenium-based SAMs [6] on Au(111). Regarding the use of an acetyl-protected SAM precursor, i.e., Trp1SeAc for SAM formation, it can be noted that the acetyl group has been cleaved from Trp1SeAc, also leading to the formation of a selenolate SAM. This finding has been reported in earlier publications on preparation of selenium-anchored SAMs from selenoacetates [5,8]. The measured thicknesses of the triptycene-based films clearly indicate that they are monolayers. Interestingly, the two thiolate SAMs are significantly thinner than the estimated maximum theoretical monolayer thicknesses, determined by the size of the Trp0S and Trp1S thiolate molecules (compare Figure 3). For less bulky organic moieties, such a finding often points to a noticeable tilt of the molecules in the SAM. However, due to the bulkiness of the triptycene unit, a tilt of its main molecular axis as defined in Figure 1 has only a small impact on the layer thickness. Oddly, since the shape of the molecules is slightly "broader" than "high" (based on the direction of the main axis, see Figure 1), a tilt of β = 0° would result in a somewhat lower layer thickness than a tilt of, e.g., 44° (the experimental value found for Trp0S). Since this difference only amounts to ca. 0.1 nm, the molecular tilt can be ruled out as the reason for the low apparent layer thicknesses of Trp0S and Trp1S. The thicknesses of the Trp0Se and Trp1Se SAMs are markedly closer to the respective theoretical maximum monolayer thicknesses than the ones of their sulfur-based analogues, while the respective tilts are quite similar (41° in Trp0Se vs 44° in Trp0S and 33° in Trp1Se vs 37° in Trp1S, see Table 3). Higher defect densities and increasing numbers of domain boundaries not closely covered with thiolate molecules cause a lower overall carbon intensity in the XP spectra – as has been found in the present study – and thus apparent lower film thicknesses. This finding lets us conclude that the nature of the anchor group is the reason for the differences in the experimental thicknesses. Selenium-based aromatic SAMs have been reported to be substantially better ordered and to have larger domains and lower defect densities than their sulfur-based analogues. The most prominent example likely is anthraceneselenolate [5] vs anthracenethiolate [26] on Au(111). The selenolate anchoring group is believed to experience a smaller corrugation of the Au(111) interaction potential, enabling it to slightly shift position and by this adapt to the sterical demand of the organic moiety of the SAM-forming molecule [5]. The thiolate, on the other hand, is confined to a specific position on the Au(111) lattice. Mismatch of periodicity of the thiolate binding position on the substrate with the sterical demands of the organic moiety causes a higher defect density and smaller domain sizes than in the anthraceneselenolate SAM [5]. This might also apply to thiolate SAMs with a triptycene-based moiety which is even bulkier than an anthracene moiety. Hence, the most plausible explanation for the smaller apparent layer thicknesses of the Trp0S and Trp1S SAMs in comparison to their selenolate counterparts is that the latter are better ordered and have lower defect densities due to the selenium anchor group.

Another feature worth discussing is the tilt angle of the triptycene-based SAMs. Note that the values of the tilt angles β as compiled in Table 3 are unambiguous due to the three-fold symmetry of the triptycene unit (see Results of NEXAFS spectroscopy and the Supporting Information File 1). In all investigated SAMs, the molecules are noticeably tilted. Interestingly, the Trp0S and Trp0Se SAMs exhibit significantly higher tilt angles (44° and 41°, respectively) than the Trp1S and Trp1Se SAMs (37° and 33°). Considering the bulkiness of the triptycene moiety, this seems on first sight rather surprising. The maximum possible tilt angle of a triptycene-based molecule adsorbed onto gold is determined by the (steric) contact of the triptycene unit with the gold surface. The closer the triptycene moiety is to the surface, the smaller should be β, if interaction with the surface dictates the degree of tilt in the SAMs. Following this argument, the Trp0S and Trp0Se SAMs are expected to be less tilted than the Trp1S and Trp1Se SAMs with their bridging –CH_2_– group. Instead, the former exhibit larger tilts. Thus, steric interaction between the triptycene moiety and the gold surface cannot be the driving force of the tilt in the investigated monolayers. Yet, the orientation of the molecules in the SAMs might also be affected by steric interaction between neighboring triptycene moieties. Due to the shape of the triptycene unit (see above discussion of the layer thickness), tilted molecules require slightly less space on the substrate surface. They can form more densely packed monolayers than non-tilted molecules and thus a tilt is energetically favored. From our point of view, this is the most likely explanation for the rather high molecular tilt in all investigated SAMs. However, what remains to be elucidated is the difference in the tilt of the Trp0S/Se SAMs (β > 40°) and the Trp1S/Se SAMs (β < 40°). Here, the bending potential of the gold–sulfur/selenium–carbon bond angle has to be considered as an additional factor. In thiolate SAMs on Au(111) with alkyl chain backbones, the gold–sulfur–carbon bond angle markedly deviates from 180°. As a consequence, the terminal groups of the molecules in a SAM are more upright in presence of an odd number of methylene spacers and are stronger tilted when an even number of –CH_2_– groups is present (or when –CH_2_– groups are absent) [10,46–48]. For instance, biphenyl- [11,39] and terphenyl-terminated [13] thiolate SAMs on gold show distinctive corresponding odd–even effects. For SAMs of similar, biphenyl-based selenolates on Au(111), the same effect has been reported [12,49–50]. As an approach to rationalize the non-linearity of the gold–sulfur–carbon bond angle, the sulfur atom's tendency of sp^3^ hybridization was discussed [13,39] and also examined in a theoretical study [51]. A bending of the gold–chalcogen–carbon angle in case of the triptycene-based SAMs studied here would result in markedly tilted Trp0S/Se SAMs, whereas a methylene group between the chalcogen and the triptycene moiety would allow for a less tilted orientation of the latter, as has been found in the present study. This strongly suggests that the concept of the gold–chalcogen–carbon angle bending potential and the odd–even effect based on it can be extended to the triptycene-based SAMs, underlining their universal character.

Finally, the thermal desorption behavior of the triptycene-based SAMs should be addressed. For the triptycene-based SAMs, Table 4 lists temperatures and activation energies of desorption. Note that the latter can serve as upper limits of thermodynamic binding energies. Interestingly, both the nature of the anchor group and the methylene spacer group have an impact on the thermal stability of the monolayers. The selenolate SAMs are more stable than their thiolate counterparts (by 0.13 eV in case of Trp0Se vs Trp0S and by ≈0.06 eV in case of Trp1Se vs Trp1S). This finding reflects that the Se–Au binding energy is higher than the one of the S–Au bond, as has been found in many studies on analogous thiolate and selenolate SAMs [8,22–25]. Insertion of a methylene spacer group into the triptycene-based SAMs even has a greater stabilizing effect than the one caused by exchanging the sulfur with the selenium anchor group: The desorption energies of the Trp1S and Trp1Se SAMs are 0.16 eV and ≈0.09 eV greater than the ones of Trp0S and Trp0Se, respectively. Hence, by releasing the steric stress exerted by the gold–chalcogen–carbon bending potential, the –CH_2_– spacer allows for an energetically more stable arrangement of the molecules in the monolayers.

In addition to the monolayers' energetic stability, their desorption pathways are worth to be discussed. Selenium–carbon bond cleavage is the main, if not the only pathway in case of Trp0Se. No evidence was found for other fragments than Trp0^+^. This fragment appears at a single temperature, indicating a monolayer with only one adsorption site (or adsorption sites with equal binding energies), and a low defect density. Comparison to the Trp1Se SAM reveals an important difference: Here the appearance of Trp1Se^+^ fragments points to an additional desorption channel with cleavage of the gold–selenium bond. The strong Se^+^ signal in the TD spectrum of the Trp1Se SAM suggests that also Se–C bond cleavage occurs during thermal desorption. Since the ionization efficiencies of the various fragments in the mass spectrometer might be different and because of possible fragmentation during the detection in the spectrometer, it is not possible to identify a main desorption channel. While for both selenolate SAMs, there is evidence for Se–C bond cleavage upon desorption, the thiolate SAMs seem to desorb as complete molecules. The appearance of (M-S)^+^, i.e., Trp0^+^ and Trp1^+^ fragments in the respective TD spectra of the Trp0S and Trp1S SAMs can most likely be explained by fragmentation of the intact thiolates during detection in the mass spectrometer, since the desorption temperatures of the M^+^ and the (M-S)^+^ signals were found to be identical. Thus, it can be tentatively concluded that desorption of both thiolate SAMs proceeds only via the intact molecules. The difference in the desorption behavior of thiolates and selenolates is in line with the assumption that the selenium–gold bond is stronger than the sulfur–gold bond (see discussion above). The strong selenium–gold bond causes a weakening of the selenium–carbon bond and its cleavage during thermal desorption of the selenolate SAMs. Similar results have been recently reported in a study of the bond-stability in naphthalene-based thiolate and selenolate SAMs on gold [8].

## Conclusion

As the systematic investigation of four triptycene-based SAMs on gold clearly reveals, the key factor that influences the tilt in the triptycene-based SAMs is the interplay of neighboring molecules, not the interaction of the triptycene moiety with the gold surface. The latter has been described in the literature as an example for the limit of weak interaction with an inorganic surface [52]. This supports the assumption that intermolecular packing is the main driving force for the strong tilt of the triptycene-based molecules in the monolayers. The gold–chalcogen–carbon bending potential forces the molecules in the Trp0S/Se SAMs into a tilt greater than 40°. The methylene unit in the Trp1S/Se SAMs enables the triptycene moieties to adopt the energetically most favorable orientation, featuring a tilt still noteworthy yet below 40°. This is reflected by the higher thermal stabilities of the Trp1S/Se SAMs compared to the ones of the Trp0S/Se monolayers. This difference is even larger than the difference of the desorption energies of the selenolate SAMs vs the respective thiolate SAMs.

Still, the anchor group plays an important role. We have found clear evidence that triptycene-based selenolate SAMs are better ordered and exhibit a lower defect density than the analogous thiolate SAMs. Domain size and, correspondingly, defect density might have a significant influence, e.g., on the usability of SAMs as templates for the growth of SURMOFs, given the potential of defects to act as nucleation sites. In this context, it will be interesting to repeat liquid phase epitaxy experiments of SURMOFs on –COOH terminated triptycene-based SAMs [20] with a selenium anchor group instead of a sulfur anchor group to study the impact of a higher degree of order on the resulting MOF thin films and their crystallographic orientation. Deposition temperature has been identified to be an important parameter in SURMOF liquid epitaxial growth [3,21]. The higher thermal stability of the selenium anchor group compared to the one of the sulfur anchor group enables triptycene-selenolate SAMs to push the upper temperature limit available for SURMOF growth. We believe that these properties specific of triptycene-based selenolate SAMs make them attractive for use as templates in the preparation of MOF thin films.

## Experimental Methods

### Synthesis of triptycene-based sulfur- and selenium compounds

We adopted literature-known protocols to synthesize Trp0SH [28], Trp1SH [28] and Trp0SeH [29]. The synthesis route for Trp1SeAc was newly developed in the doctoral thesis of one of us (B. S.) [30] and is presented here for a first time in a scientific journal. The synthesis starts from 9-(chloromethyl)anthracene (see Scheme S1 and experimental details of the synthesis route in the Supporting Information File 1).

### Substrates with Au(111) surfaces

For the investigation of the SAMs with infrared spectroscopy and electron spectroscopy, single-crystalline Si(100) wafer (Wacker) pieces with evaporated gold layers were used as substrates. Metal deposition was carried out using a commercial vaporisator (Leybold Univex 300). Gold (Chempur, 99.995%) layers with a thickness of 150 nm were deposited at a rate of 1 nm/s. An 8 nm titanium (Chempur, 99.8%) layer was deposited at a rate of 0.15 nm/s as an adhesion layer between the Si substrate and the Au layer. The deposition rate and thickness were monitored using a quartz crystal microbalance.

Substrates for thermal desorption spectroscopy measurements were gold covered mica sheets. Freshly cleaved mica sheets (Mahlwerk Neubauer - Friedrich Geffers) were heated to 280 °C for about two days inside the evaporation chamber to remove residual water and other contaminations from the ambient. Subsequently, a 140 nm gold layer (99.995%, Chempur) was deposited by thermal evaporation at a substrate temperature of 280 °C and a pressure of ≈10^−7^ mbar using the above-mentioned vaporisator. The substrate was cooled down to room temperature in the evaporation chamber after deposition.

### Preparation of monolayers

The Trp0S, Trp0Se, Trp1S and Trp1Se SAMs were prepared by immersing Au substrates into ≈20 µM ethanolic solutions of the corresponding triptycene-based compounds for 20–24 h at room temperature. After removal of the samples from solution, they were rinsed with ethanol and dried in a stream of N_2_.

### Infrared spectroscopy

Spectra of KBr pellets containing the triptycene-based compounds were recorded at room temperature using a dry-air purged BioRad Excalibur FTS-3000 Fourier-transform infrared spectrometer equipped with a deuterated triglycine sulfate detector. Infrared-reflection–absorption (IRRA) spectra of the SAMs were taken with an ultra-high vacuum apparatus (Prevac) with an attached FTIR spectrometer (Bruker VERTEX 80v) which has been described elsewhere [53]. The base pressure of the measurement chamber was 2 × 10^−10^ mbar.

All IRRA spectra were recorded in grazing incidence reflection mode at an incidence angle of 80° relative to the surface normal using a liquid nitrogen cooled mercury cadmium telluride narrow band detector. Perdeuterated hexadecanethiolate-SAMs on gold-covered silicon wafer pieces were used for reference measurements.

All spectra were acquired at a resolution of 2 cm^−1^.

### Calculation of IR spectra

Theoretical values of the vibrational frequencies of the isolated molecules were obtained employing quantum-chemical density functional theory (DFT) calculations with the Gaussian 03 program package [54], using the B3LYP hybrid density functional [55–58] and the cc-pVDZ basis set [59]. The computed IR-frequencies were scaled by a factor of 0.9758. This factor was obtained by comparing the calculated and the experimental value of the band of an intense triptycene vibrational mode (calculated value: 1488 cm^−1^, experimentally found: 1452 cm^−1^). The triptycene spectrum was taken from the NIST database [60]. The calculated spectra were used to aid the assignment of the vibrational bands and to estimate the direction of the corresponding transition dipole moments (TDMs).

### XPS

Note that due to the limitations of the accessible XPS instrumentation, it was not possible to obtain S 2p and Se 3p photoelectron spectra. The purpose of the XP spectra was the evaluation of the Au 4f and the C 2s resonances to obtain layer thicknesses [34]. X-ray photoelectron spectroscopy (XPS) measurements were performed at room temperature using two UHV apparatuses based on modified Leybold XPS systems with double-anode X-ray sources (Al Kα). The base pressure of the analyzing chambers amounted to 10^−10^ mbar. The samples were irradiated at normal incidence. The energy resolutions were 0.8 eV (in case of the Trp0S and Trp0Se samples) and 1.1 eV (in case of the Trp1S and Trp1Se), respectively. The energy scales of all spectra were referenced to the Au 4 f_7/2_ peak located at a binding energy of 84.0 eV. To evaluate the layer thicknesses of the investigated SAMs, the samples were mounted on a holder together with a reference of well-known-thickness, a *n*-decanethiolate SAM on Au(111) [61], thus ensuring identical geometric conditions (i.e., distance and angles of X-ray gun and energy analyzer toward the sample) for sample and reference.

### TDS

Thermal desorption spectroscopy (TDS) experiments were performed in a UHV apparatus with a base pressure of 10^−10^ mbar which has been described in detail elsewhere [62]. A quadrupole mass spectrometer (Balzers QMS200 quadrupole, mass range 0–300 amu) with a Feulner cup was used to record the thermal desorption spectra. The samples were heated with a linear ramp of β_T_ = 0.5 K/s.

### NEXAFS spectroscopy

The near-edge X-ray absorption fine structure (NEXAFS) measurements were performed at the dipole beamline HE-SGM of the synchrotron storage ring BESSY II in Berlin (Germany). Spectral acquisition was carried out at the C K-edge in the partial electron yield mode with a retarding voltage of −150 V with a linear polarization factor *P* ≈ 91%. Energy resolution was better than 350 meV. During measurements, the samples were kept at room temperature in the analytical chamber with a base pressure of ≈10^−9^ mbar. No damage of the samples was observed.

The NEXAFS raw data were normalized in a multi-step procedure considering the incident photon flux by division by a spectrum of a clean, freshly sputtered Au substrate. The energy was scaled using the signal of a carbon contamination of a gold grid with a characteristic peak at 284.81 eV. To obtain the molecular orientation of the thiolates/selenolates relative to the substrate surface, spectra were taken at different incidence angles of the synchrotron radiation (20°, 30°, 55°, 70° and 90° with respect to the surface).

## Supporting Information

File 1 Supporting information includes experimental details on the synthesis of Trp1SeAc, an extended table with IR spectra band assignments, original NEXAFS spectra and the derivation of Equation 3 and Equation 4.

## References

[1] Love J C, Estroff L A, Kriebel J K, Nuzzo R G, Whitesides G M (2005). Chem Rev.

[2] Kind M, Wöll C (2009). Prog Surf Sci.

[3] Zhuang J-L, Terfort A, Wöll C (2016). Coord Chem Rev.

[4] Schreiber F (2000). Prog Surf Sci.

[5] Bashir A, Käfer D, Müller J, Wöll C, Terfort A, Witte G (2008). Angew Chem, Int Ed.

[6] Romashov L V, Ananikov V P (2013). Chem – Eur J.

[7] Hohman J N, Thomas J C, Zhao Y, Auluck H, Kim M, Vijselaar W, Kommeren S, Terfort A, Weiss P S (2014). J Am Chem Soc.

[8] Ossowski J, Wächter T, Silies L, Kind M, Noworolska A, Blobner F, Gnatek D, Rysz J, Bolte M, Feulner P (2015). ACS Nano.

[9] Bain C D, Troughton E B, Tao Y T, Evall J, Whitesides G M, Nuzzo R G (1989). J Am Chem Soc.

[10] Tao Y T, Lee M T, Chang S C (1993). J Am Chem Soc.

[11] Azzam W, Cyganik P, Witte G, Buck M, Wöll C (2003). Langmuir.

[12] Cyganik P, Szelagowska-Kunstman K, Terfort A, Zharnikov M (2008). J Phys Chem C.

[13] Shaporenko A, Brunnbauer M, Terfort A, Grunze M, Zharnikov M (2004). J Phys Chem B.

[14] Azzam W, Bashir A, Terfort A, Strunskus T, Wöll C (2006). Langmuir.

[15] Dameron A A, Charles L F, Weiss P S (2005). J Am Chem Soc.

[16] Kim M, Hohman J N, Morin E I, Daniel T A, Weiss P S (2009). J Phys Chem A.

[17] Hohman J N, Kim M, Schüpbach B, Kind M, Thomas J C, Terfort A, Weiss P S (2011). J Am Chem Soc.

[18] Saavedra H M, Barbu C M, Dameron A A, Mullen T J, Crespi V H, Weiss P S (2007). J Am Chem Soc.

[19] Dameron A A, Mullen T J, Hengstebeck R W, Saavedra H M, Weiss P S (2007). J Phys Chem C.

[20] Liu J, Shekhah O, Stammer X, Arslan H K, Liu B, Schüpbach B, Terfort A, Wöll C (2012). Materials.

[21] Zhuang J-L, Kind M, Grytz C M, Farr F, Diefenbach M, Tussupbayev S, Holthausen M C, Terfort A (2015). J Am Chem Soc.

[22] Huang F K, Horton R C, Myles D C, Garrell R L (1998). Langmuir.

[23] Sato Y, Mizutani F (2004). Phys Chem Chem Phys.

[24] Shaporenko A, Cyganik P, Buck M, Terfort A, Zharnikov M (2005). J Phys Chem B.

[25] Szelągowska-Kunstman K, Cyganik P, Schüpbach B, Terfort A (2010). Phys Chem Chem Phys.

[26] Käfer D, Witte G, Cyganik P, Terfort A, Wöll C (2006). J Am Chem Soc.

[27] Dauselt J, Zhao J, Kind M, Binder R, Bashir A, Terfort A, Zharnikov M (2011). J Phys Chem C.

[28] Fowelin C, Schüpbach B, Terfort A (2007). Eur J Org Chem.

[29] Ishii A, Matsubayashi S, Takahashi T, Nakayama J (1999). J Org Chem.

[30] Schüpbach B (2013). Synthese von Arylalkanthiolen zur Darstellung gezielt funktionalisierter Oberflächen.

[31] Greenler R G (1966). J Chem Phys.

[32] Wesner D A, Coenen F P, Bonzel H P (1988). Phys Rev Lett.

[33] Bashir A, Heck A, Narita A, Feng X, Nefedov A, Rohwerder M, Müllen K, Elstner M, Wöll C (2015). Phys Chem Chem Phys.

[34] Dannenberger O, Weiss K, Himmel H-J, Jäger B, Buck M, Wöll C (1997). Thin Solid Films.

[35] Hansen H S, Tougaard S, Biebuyck H (1992). J Electron Spectrosc Relat Phenom.

[36] Bain C D, Whitesides G M (1989). J Phys Chem.

[37] Himmel H-J (1998). Strukturen und Reaktivitäten von selbstorganisierenden Dünnstschichten aus Organothiolen.

[38] Bagus P S, Weiss K, Schertel A, Wöll C, Braun W, Hellwig C, Jung C (1996). Chem Phys Lett.

[39] Rong H-T, Frey S, Yang Y-J, Zharnikov M, Buck M, Wühn M, Wöll C, Helmchen G (2001). Langmuir.

[40] Ballav N, Schüpbach B, Dethloff O, Feulner P, Terfort A, Zharnikov M (2007). J Am Chem Soc.

[41] Liu J, Schüpbach B, Bashir A, Shekhah O, Nefedov A, Kind M, Terfort A, Wöll C (2010). Phys Chem Chem Phys.

[42] Käfer D, Wöll C, Witte G (2009). Appl Phys A.

[43] Oja V, Suuberg E M (1998). J Chem Eng Data.

[44] Chickos J S, Hanshaw W (2004). J Chem Eng Data.

[45] Ulbricht H, Zacharia R, Cindir N, Hertel T (2006). Carbon.

[46] Walczak M M, Chung C, Stole S M, Widrig C A, Porter M D (1991). J Am Chem Soc.

[47] Laibinis P E, Whitesides G M, Allara D L, Tao Y T, Parikh A N, Nuzzo R G (1991). J Am Chem Soc.

[48] Chang S-C, Chao I, Tao Y-T (1994). J Am Chem Soc.

[49] Shaporenko A, Müller J, Weidner T, Terfort A, Zharnikov M (2007). J Am Chem Soc.

[50] Weidner T, Shaporenko A, Müller J, Schmid M, Cyganik P, Terfort A, Zharnikov M (2008). J Phys Chem C.

[51] Heimel G, Romaner L, Brédas J-L, Zojer E (2008). Langmuir.

[52] Fernandez-Torrente I, Franke K J, Henningsen N, Schulze G, Alemani M, Roth C, Rurali R, Lorente N, Pascual J I (2006). J Phys Chem B.

[53] Wang Y, Glenz A, Muhler M, Wöll C (2009). Rev Sci Instrum.

[54] (2004). Gaussian 03.

[55] Becke A D (1988). Phys Rev A.

[56] Lee C, Yang W, Parr R G (1988). Phys Rev B.

[57] Stephens P J, Devlin F J, Chabalowski C F, Frisch M J (1994). J Phys Chem.

[58] Becke A D (1993). J Chem Phys.

[59] Dunning T H (1989). J Chem Phys.

[60] Linstrom E P J, Mallard W G (2016). NIST Chemistry WebBook, NIST Standard Reference Database Number 69.

[61] Biebuyck H A, Bain C D, Whitesides G M (1994). Langmuir.

[62] Loepp G, Vollmer S, Witte G, Wöll C (1999). Langmuir.

