# Supplementary material for: Triptycene-terminated thiolate and selenolate monolayers on Au(111)
Source: Beilstein J Nanotechnol. 2017 Apr 20;8:892–905. doi: 10.3762/bjnano.8.91 (PMC5405688; doi:10.3762/bjnano.8.91)
Supplement: File 1 — Supporting information includes experimental details on the synthesis of Trp1SeAc, an extended table with IR spectra band assignments, original NEXAFS spectra and the derivation of Equation 3 and Equation 4. [file Beilstein_J_Nanotechnol-08-892-s001.pdf]

Supporting Information

for

Triptycene-terminated thiolate and selenolate monolayers on Au(111)

Jinxuan Liu[1], Martin Kind*[2], Björn Schüpbach[2], Daniel Käfer[3], Stefanie Winkler[3], Wenhua Zhang[4], Andreas Terfort[2] and Christof Wöll*[5]

Address: [1]Institute of Artificial Photosynthesis, State Key Laboratory of Fine Chemicals, Dalian University of Technology, 116024 Dalian, China [2]Institut für Anorganische und Analytische Chemie, Goethe-Universität Frankfurt am Main, 60325 Frankfurt, Germany, [3]Physikalische Chemie I, Ruhr-Universität Bochum, 44780 Bochum, Germany, [4]National Synchrotron Radiation Laboratory, University of Science and Technology of China, Hefei, Anhui 230029, China and [5]Institute of Functional Interfaces, Karlsruhe Institute of Technology (KIT), 76021 Karlsruhe, Germany

Email: Martin Kind - kind@chemie.uni-frankfurt.de, Christof Wöll - christof.woell@kit.edu

* Corresponding author

Supporting information includes experimental details on the synthesis of Trp1SeAc, an extended table with IR spectra band assignments, original NEXAFS spectra and the derivation of Equation 3 and Equation 4.

Experimental details on the synthesis of Trp1SeAc

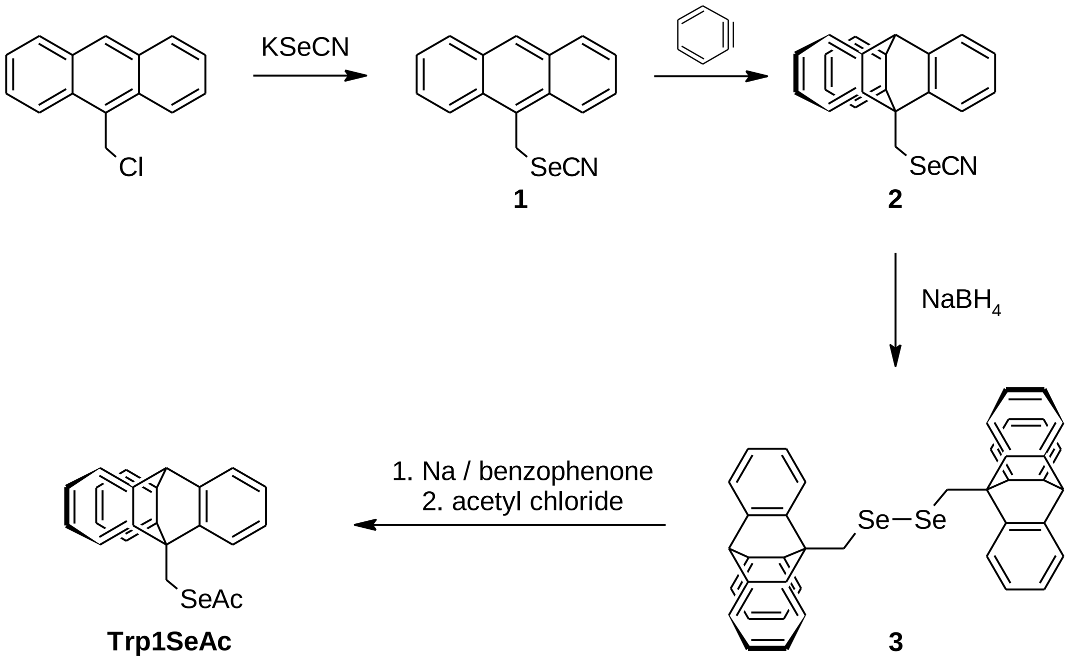

Scheme S1: Synthetic route from 9-chloromethylanthracene to (9-triptycene)methyl selenoacetate.

This information has already been published in part in ref. [1]. If not indicated otherwise, the synthesis steps were carried out under inert gas in Schlenk apparatuses.

9-Anthrylmethylselenocyanate (1). 9-Chloromethylanthracene (7.11 g, 31.5 mmol) was dissolved in 120 ml abs. dimethylformamide. Potassium selenocyanate (5.19 g, 36.0 mmol) was added under external cooling with ice water. After stirring the solution for 2 hours at room temperature, the solvent was removed in vacuum. The residue was dissolved in water and dichloromethane, the phases were separated and the organic phase was concentrated. The raw product was filtrated through silica gel and recrystallized from chloroform. 9-Anthrylmethylselenocyanate **1** was obtained as a yellow solid (6.22 g, 21.0 mmol, yield = 67%), mp = 157 °C (dec.) ^{1}H NMR ($CDCl_3$, 400 MHz): H: δ=8.50 (s, 1H, H-10), 8.25 (d, 2H, ^{3}J = 8.8 Hz, H-4, H-5), 8.05 (d, 2H, ^{3}J = 8.5 Hz, H-1, H-8), 7.70-7.86 (m, 2H, H-3, H-6), 7.57-7.49 (m, 2H, H-2, H-7), 5.49 (s, 2H, CH_2), ppm. ^{13}C NMR ($CDCl_3$, 100 MHz): δ=131.4 (arom. C), 129.9 (arom. C), 129.6 (arom. C), 129.6 (arom. C-H), 127.3 (arom. C-H), 125.5

(arom. C-H), 123.0 (arom. C-H), 102.2 (SeCN), 26.7 (CH_2) ppm. ^{77}Se NMR ($CDCl_3$, 57 MHz): δ=227.5 ppm. Elemental analysis: $C_{16}H_{11}NSe$ (296.23): calculated: C 64.87, H 3.74, N 4.73; found: C 64.69, H 3.58, N 4.65.

9-Triptycylmethylselenocyanate (2). Anthranilic acid (5.31 g, 38.7 mmol) and five drops of trifluoroacetic acid were dissolved in 40 ml tetrahydrofuran. Amyl nitrite (6.97 g, 46.6 mmol) was added to the stirred solution under ambient conditions. 2-diazoniumphenylcarboxylate formed as a brown precipitate, which was filtered from the mother solution and washed with a small portion of tetrahydrofuran (warning: 2-diazoniumphenylcarboxylate is explosive in absence of the solvent). The thus obtained crude diazonium salt was suspended in dioxane. Substance **1** (3.16 g, 10.6 mmol) was dissolved in 60 ml of dioxane and the solution was heated to 75 °C. To the solution, 2-diazoniumphenylcarboxylate suspension in dioxane was added in small portions. Afterwards, the mixture was stirred 1 hour at 75 °C. After removal of the solvent, the tar-like residual was filtrated through silica gel using dichloromethane as eluent. The resulting raw product was purified by silica gel chromatography using a 4:1 petroleum ether-dichloromethane gradient and recrystallized from ethanol. 9-Triptycylmethylselenocyanate **2** (yield: 2.24 g, 6.02 mmol, 57%) was obtained as a light yellow solid, mp = 183-184 °C. ^1H NMR ($CDCl_3$, 250 MHz): δ=7.55-7.33 (m, 6H, H-1, H-4, H-5, H-8, H-11, H-14), 7.18-7.00 (m, 6H, H-2, H-3, H-6, H-7, H-12, H-13), 5.41 (s, 1H, H-10), 4.80 (s, 2H, CH_2) ppm. ^{13}C NMR ($CDCl_3$, 75 MHz): δ=125.9 (arom. C-H), 125.2 (arom. C-H), 124.1 (arom. C-H), 103.1 (SeCN), 54.0 (C-Trp), 53.1 (C-Trp), 29.5 (CH_2) ppm. ^{77}Se NMR ($CDCl_3$, 57 MHz): δ=169.7 ppm. MS (EI): *m/z* = 267 (100%, $[TrpCH_2]^+$), 252 (87%, Trp^+), 346 (40%, $[Trp\text{-}CHSe]^+$), 373 (25%, M^+). Elemental analysis: $C_{22}H_{15}NSe$ (372.32): calculated: C 70.97, H 4.06, N 3.76; found: C 70.77, H 3.89, N 3.72.

Bis-(triptycenylmethyl)-diselenide (3). Substance **2** (2.70 g, 7.25 mmol) and sodiumborohydride (0.80 g, 21 mmol) were dissolved in 200 ml ethanol and refluxed for 1 hour. The residue was dissolved in a mixture of dichloromethane and hydrochloric acid

(20%). After phase separation, the solvent was removed from the organic phase and the residue was recrystallized from toluene to yield bis-(triptycenylmethyl)-diselenide **3** (0.79 g, 1.1 mmol, 31%) as a yellow solid, mp = 184-185 °C. ^{1}H NMR ($CDCl_3$, 250 MHz): δ=8.10-7.60 (s, 3H, H-Trip.), 7.58-7.40 (m, 3H, H-Trip.), 7.23-7.03 (m, 3H, H-Trip.), 5.46 (s, 1H, H-10), 5.07 (s, 2H, CH_2) ppm. ^{13}C NMR ($CDCl_3$, 75 MHz): δ=146.5 (brd. s, arom. C), 125.4 (arom. C-H), 124.9 (arom. C-H), 123.7 (arom. C-H), 55.1 (C-Trp), 54.2 (C-Trp), 32.8 (CH_2) ppm. ^{77}Se NMR ($CDCl_3$, 57 MHz): δ=338.1 ppm. Elemental analysis: $C_{42}H_{30}Se_2$ (692.61): calculated: C 72.83, H 4.37; found: C 70.72, H 4.11.

(9-triptycene)methyl selenoacetate (Trp1SeAc). To a solution of **3** (0.2 g, 0.29 mmol) in 10 ml tetrahydrofuran, ca. 60 mg shredded Na and a spatula tip of benzophenone were added. During refluxing for 2 hours, the solution turned violet. Using a syringe, the solution was transferred to a beaker with 1 ml of acetyl chloride under ambient conditions. The mixture turned colorless immediately. After neutralization by addition of saturated aqueous $NaHCO_3$ solution, the mixture was extracted with dichloromethane. The solvent was removed and the residual was chromatographed over silica gel using a 5:1 petroleum ether/dichloromethane gradient. 9-Triptycenylmethylselenoacetate **Trp1SeAc** (0.11 g, 2,8 mmol, 49%) was obtained as a colorless solid, mp = 315-317 °C. ^{1}H NMR ($CDCl_3$, 250 MHz): δ=7.45-7.33 (m, 6H, H-1, H-4, H-5, H-8, H-11, H-14), 7.08-6.96 (m, 6H, H-2, H-3, H-6, H-7, H-12, H-13), 5.37 (s, 1H, H-10), 4.50 (s, 2H, CH_2), 2.60 (s, 3H, CH_3) ppm. ^{13}C NMR ($CDCl_3$, 62 MHz): δ=197.9 (C=O), 125.3 (arom. C-H), 124.8 (arom. C-H), 123.5 (arom. C-H), 54.3 (C-9), 53.2 (C-10), 34.7 (CH_2), 24.5 (CH_3) ppm. ^{77}Se NMR ($CDCl_3$, 57 MHz): δ=524.1 ppm. MS (EI): *m/z* = 265 (100%), 346 (40%, $[Trp\text{-}CHSe]^+$), 390 (11%, M^+). Elemental analysis: $C_{23}H_{18}OSe$ (389.35): calculated: C 70.95, H 4.66; found: C 70.90, H 4.54.

Extended IR spectra band assignment

Table S1: Band assignments of triptycene-terminated species as obtained from the calculated, KBr pellet and SAM spectra along with the orientation of their transition dipole moments (TDMs) relative to the main molecular axis as deduced from the calculations.

			Band position / cm^{-1}											
No	Assignment[1]	TDM[2]	Trp0S			Trp0Se			Trp1S			Trp1Se		
			Calc.	KBr	SAM	Calc.	KBr	SAM	Calc.	KBr	SAM	Calc.	KBr	SAM
1	ν CH arom	_|_	3129	3068	3069	3123	3066	3066	3127	3058	3069	3123	3069	3070
2	ν CH arom		3118	3038		3118	3036		3117	3033	3047	3116	3036	3047
3	ν CH arom		3103	3021	3021	3103	3012	3012	3102	3020	3018	3103	3017	3018
4	ν CH aliph	||	3030	2950	2962	3026	2950	2963	3029	2972	2960	3028	2978	2961
5	ν CH_2 as	_|_							2988	2933	2926	2992	2931	2930
6	ν CH_3 s											2981	2913	2907
7	ν SH / ν SeH		2606	2569		2315	2310		2598	2562				
8	ν CO											1774	1710	
9	δ CH arom	_|_, ||[3]	1450	1455	1456	1449	1456	1455	1545	1457	1457	1451	1457	1457
10	δ CH arom	_|_	1442	1450	1444	1442	1448	1442[4]						
11	δ CH_2	/							1428	1431	1414	1419	1429	1414
12	δ CH_3 as											1398	1409	
13	δ CH arom	_|_	1276	1287	1285	1278	1287	1286	1290	1319	1322			
14	δ CH arom	||	1268	1287	1285	1269	1287	1288	1281	1301	1296			
15	γ CH_2	/							1255	1266	1241	1226	1236	1236
16	ν CC	||							1180	1189	1186			
17	δ CH ν CC arom	_|_	1161	1169	1150	1160	1156	1157						
18	δ CH arom	||	1141	1146	1135	1139	1145	1148	1129	1133	1130	1123	1134	
19	ν CC ρ CH_3											1088	1109	
20	ν CC ρ arom	||	1032	1035	1036	1032	1035	1027	1033	1037	1049	1033	1036	1034
21	νoν CSe	||	937	961	940	917	939	920						
22	ρ CH_3											928	950	

1) ν: stretching, δ: bending, γ: wagging, ρ: rocking

arom: aromatic, aliph: aliphatic, s: symmetric, as: asymmetric

2) _|_: perpendicular or almost perpendicular to main molecular axis, ||: parallel or almost parallel to main molecular axis, /: neither parallel nor perpendicular to main molecular axis

3) several unresolved modes

4) shoulder

Original NEXAFS spectra

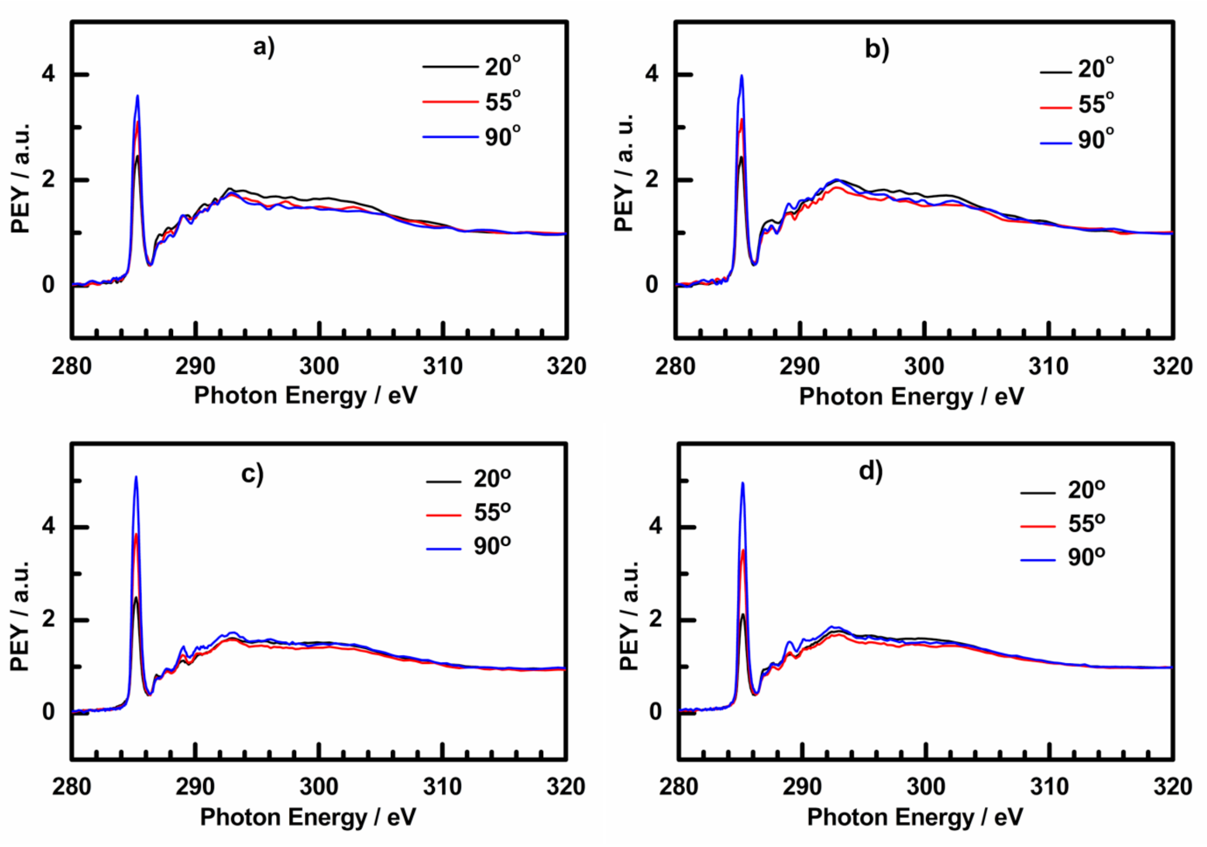

Figure S1: NEXAFS spectra of (a) Trp0S, (b) Trp0Se, (c) Trp1S, and (d) Trp1Se SAMs on Au(111).

Derivation of equations (3) and (4)

This section intends to derive the relationship between the NEXAFS C 1s-π_1* transition absorption signal dichroism and the tilt β of the molecular main axes of the triptycene-based molecules against the substrate surface normal. We start with defining the main molecular axis **n** in the triptycene unit that passes through both aliphatic C atoms and represents the line of intersection of all three aromatic planes contained in the triptycene unit (compare Figure S2). Presuming independent C 1s-π_1* transitions for each of the three aromatic rings in the triptycene unit, three transition dipole moments $\mathbf{t_1}$, $\mathbf{t_2}$, and $\mathbf{t_3}$ result, that are all perpendicular to **n** and rotated against each other by 120° steps. Let us initially concentrate on one of these transition dipole moment vectors, e.g. $\mathbf{t_1}$, whose angle with the surface normal

shall be defined as α. The angles α and β are connected via a third angle γ that represents the rotation of the molecule about the axis **n**.

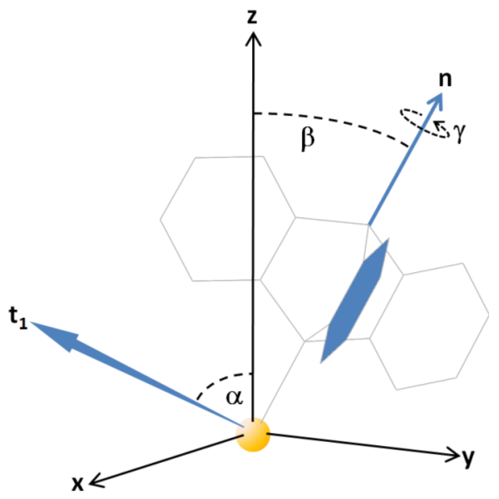

Figure S2: Schematic drawing of a triptycene-based molecule in the investigated SAMs. The chalcogen atom is located in the origin of the coordinate system, the z-axis represents the substrate surface normal. The main axis **n** of the molecule is tilted against the z-axis by the angle β. One of the three aromatic rings of the triptycene unit is highlighted in blue. The transition dipole moment designated $\mathbf{t_1}$ is oriented perpendicular to this aromatic ring's plane. The vector $\mathbf{t_1}$ is tilted against the surface normal by the angle α. The relationship between α and β and the rotation of the molecule about the main axis, given by the angle γ, is defined as $\cos(\alpha) = \sin(\beta) \cdot \cos(\gamma)$. Note that two other transition dipole moments $\mathbf{t_2}$ and $\mathbf{t_3}$ (not shown in the figure) exist that are perpendicular to the planes of the respective other aromatic rings. As a consequence of the symmetry of the triptycene unit, the transition dipole moments vectors $\mathbf{t_1}$, $\mathbf{t_2}$, and $\mathbf{t_3}$ are rotated against each other by 120° steps.

We proceed by showing that the angles α, β and γ are related by the formula $\cos(\alpha) = \sin(\beta) \cdot \cos(\gamma)$ [2,3]. In a hypothetical starting orientation of the triptycene-based molecule, the chalcogen anchor atom is located in the origin and the molecule is completely upright. Hence, the starting molecular main axis **n** is oriented parallel to the surface normal **z** and is defined to have unity length, i.e. $\mathbf{n} = (0,0,1)$. The starting transition dipole moment $\mathbf{t_1}$ is oriented parallel to the y-axis and has an absolute value t, i.e. $\mathbf{t_1} = (0,t,0)$. In this starting orientation, β (tilt) and γ (rotation about the main axis) are both defined to be 0.

Subsequent rotation about the surface normal **z** by γ and about the x-axis by β transfers the molecule into a final orientation that is supposed to be existent in the monolayer. The matrix representations of the rotational operations are

$$\underline{R_z}(\gamma) = \begin{pmatrix} \cos\gamma & -\sin\gamma & 0 \\ \sin\gamma & \cos\gamma & 0 \\ 0 & 0 & 1 \end{pmatrix}$$

for the rotation about the z-axis by γ and

$$\underline{R_x}(\beta) = \begin{pmatrix} 1 & 0 & 0 \\ 0 & \cos\beta & -\sin\beta \\ 0 & \sin\beta & \cos\beta \end{pmatrix}$$

for the rotation about the x-axis by β.

The final molecular axis vector will then be

$$\boldsymbol{n_{final}} = \underline{R_x}(\beta)\underline{R_z}(\gamma)\boldsymbol{n} = \begin{pmatrix} 1 & 0 & 0 \\ 0 & \cos\beta & -\sin\beta \\ 0 & \sin\beta & \cos\beta \end{pmatrix} \begin{pmatrix} \cos\gamma & -\sin\gamma & 0 \\ \sin\gamma & \cos\gamma & 0 \\ 0 & 0 & 1 \end{pmatrix} \begin{pmatrix} 0 \\ 0 \\ 1 \end{pmatrix} = \begin{pmatrix} 0 \\ -\sin\beta \\ \cos\beta \end{pmatrix}. \tag{S1}$$

Note that regardless of the value of γ, the molecular axis is now tilted by β against the surface normal.

Applying both rotational operations to $\mathbf{t_1}$ yields

$$\boldsymbol{t_{1,final}} = \underline{R_x}(\beta)\underline{R_z}(\gamma)\boldsymbol{t_1} = t \cdot \begin{pmatrix} 1 & 0 & 0 \\ 0 & \cos\beta & -\sin\beta \\ 0 & \sin\beta & \cos\beta \end{pmatrix} \begin{pmatrix} \cos\gamma & -\sin\gamma & 0 \\ \sin\gamma & \cos\gamma & 0 \\ 0 & 0 & 1 \end{pmatrix} \begin{pmatrix} 0 \\ 1 \\ 0 \end{pmatrix} = t \cdot \begin{pmatrix} -\sin\gamma \\ \cos\beta\cos\gamma \\ \sin\beta\cos\gamma \end{pmatrix} \tag{S2}$$

for the final orientation of the transition dipole moment.

The cosine of the angle between $\mathbf{t_{1,final}}$ and the surface normal, i.e. the z-axis, can be obtained by calculating the inner product of $\mathbf{t_{1,final}}$ and **z**:

$$\cos\alpha = \boldsymbol{z} \cdot (\boldsymbol{t_{1,final}}/t) = \begin{pmatrix} 0 \\ 0 \\ 1 \end{pmatrix} \cdot \begin{pmatrix} -\sin\gamma \\ \cos\beta\cos\gamma \\ \sin\beta\cos\gamma \end{pmatrix} = \sin\beta\cos\gamma. \tag{S3}$$

The magnitude of the NEXAFS absorption signal *I* is related to the orientation of the transition dipole moment $\mathbf{t_{1,final}}$ and the electric field vector **E** of the synchrotron radiation by [4]:

$$I \propto (\boldsymbol{E} \cdot \boldsymbol{t_{1,final}})^2 \tag{S4}$$

Based on the considerations of Stöhr [4] for layers on substrate surfaces with 3-fold (or higher) symmetry, equation (S4) can be evaluated to obtain the term:

$$I = B \cdot \left\{ P \cdot \left(\cos^2\theta \cos^2\alpha + \frac{1}{2}\sin^2\theta \sin^2\alpha \right) + \frac{1-P}{2} \cdot (1 - \cos^2\theta) \right\} \tag{S5}$$

with the angle θ between the substrate surface normal **z** and the electric field vector **E**. The quantity *P* represents the degree of polarization of the synchrotron X-ray radiation as defined by Stöhr [4]. The parameter *B* combines an instrument constant with the quantity *t*. In equation 9.16 in Ref. [4], Stöhr gives a formula almost identical to (S5) but without the polarization factor *P*. When applying *P* like in the derivation of equation 9.11 in Ref. [4], equation (S5) can be derived easily.

With the help of addition theorems, equation (S5) can be rewritten:

$$I = \frac{B \cdot P}{2} \cdot (3\cos^2\alpha - 1) \cdot \cos^2\theta + \frac{B}{2} \cdot (1 - \cos^2\alpha) \tag{S6}$$

The term $\cos^2\alpha$ can be substituted using equation (S3), to yield

$$I = \frac{B \cdot P}{2} \cdot (3\sin^2\beta\cos^2\gamma - 1) \cdot \cos^2\theta + \frac{B}{2} \cdot (1 - \sin^2\beta\cos^2\gamma) \tag{S7}$$

We now consider the three-fold symmetry of the triptycene moiety and assume that for each aromatic ring, a C 1s-π_1* transition of equal intensity occurs. Their transition dipole moment vectors $\mathbf{t_1}$, $\mathbf{t_2}$, and $\mathbf{t_3}$ then have identical magnitudes *t*. In a hypothetical starting orientation of the molecule (see above), $\mathbf{t_1} = (0,t,0)$ and $\mathbf{t_2}$ and $\mathbf{t_3}$ can be obtained by rotating $(0,t,0)$ about the z-axis by 120° and 240°, respectively. Thus, after rotation about the z-axis by γ, the angles of $\mathbf{t_{1,final}}$, $\mathbf{t_{2,final}}$, and $\mathbf{t_{3,final}}$ will be γ, γ+2π/3 and γ+4π/3, respectively.

The overall NEXAFS absorption signal I_{total} is the sum of the signals of the individual aromatic rings and can be expressed as:

$$\begin{aligned}
I_{total} = \; & I(\boldsymbol{t}_{1,final}) + I(\boldsymbol{t}_{2,final}) + I(\boldsymbol{t}_{3,final}) \\
= \; & \frac{B \cdot P}{2} \cdot (3\sin^2\beta\cos^2\gamma - 1) \cdot \cos^2\theta + \frac{B}{2} \cdot (1 - \sin^2\beta\cos^2\gamma) + \\
& \frac{B \cdot P}{2} \cdot \left(3\sin^2\beta\cos^2\left(\gamma + \frac{2\pi}{3}\right) - 1\right) \cdot \cos^2\theta + \frac{B}{2} \cdot \left(1 - \sin^2\beta\cos^2\left(\gamma + \frac{2\pi}{3}\right)\right) + \\
& \frac{B \cdot P}{2} \cdot \left(3\sin^2\beta\cos^2\left(\gamma + \frac{4\pi}{3}\right) - 1\right) \cdot \cos^2\theta + \frac{B}{2} \cdot \left(1 - \sin^2\beta\cos^2\left(\gamma + \frac{4\pi}{3}\right)\right) \\
= \; & \frac{B \cdot P}{2} \cdot \left(3\sin^2\beta\left(\cos^2\gamma + \cos^2\left(\gamma + \frac{2\pi}{3}\right) + \cos^2\left(\gamma + \frac{4\pi}{3}\right)\right) - 3\right) \cdot \cos^2\theta + \\
& \frac{B}{2} \cdot \left(3 - \sin^2\beta\left(\cos^2\gamma + \cos^2\left(\gamma + \frac{2\pi}{3}\right) + \cos^2\left(\gamma + \frac{4\pi}{3}\right)\right)\right)
\end{aligned} \tag{S8}$$

Using addition theorems, it can be shown that $\cos^2(x)+\cos^2(x+2\pi/3)+\cos^2(x+4\pi/3) = 3/2$. Thus, the overall NEXAFS signal can finally be expressed as

$$I_{total} = \frac{3BP}{2} \cdot \left(\frac{3}{2}\sin^2\beta - 1\right) \cdot \cos^2\theta + \frac{3B}{2} \cdot \left(1 - \frac{1}{2}\sin^2\beta\right). \quad (3)$$

At this point, the impact of the three-fold symmetry of triptycene-based molecules on the evaluation of NEXAFS data should be discussed. First, the NEXAFS signal strength is only dependent on β, i.e. it is impossible to gain information on γ by evaluation of the NEXAFS signal. On the other hand, β can be unambiguously obtained by plotting I_{total} against $\cos^2\theta$. The resulting function is a straight line with the intercept

$$a = \frac{3B}{2} \cdot \left(1 - \frac{1}{2}\sin^2\beta\right)$$

and the slope

$$m = \frac{3BP}{2} \cdot \left(\frac{3}{2}\sin^2\beta - 1\right).$$

The tilt angle of the molecular axis then is

$$\beta = \sin^{-1}\left(\sqrt{\frac{2m+2Pa}{m+3Pa}}\right). \quad (4)$$

References

(1) Schüpbach, B. *Synthese von Arylalkanthiolen zur Darstellung gezielt funktionalisierter Oberflächen*; PhD Thesis, Frankfurt University, Frankfurt am Main, Germany, 2013.

(2) Rong, H.-T.; Frey, S.; Yang, Y.-J.; Zharnikov, M.; Buck, M.; Wühn, M.; Wöll, C.; Helmchen, G. *Langmuir* **2001**, *17* (5), 1582–1593.

(3) Ballav, N.; Schüpbach, B.; Dethloff, O.; Feulner, P.; Terfort, A.; Zharnikov, M. *J. Am. Chem. Soc.* **2007**, *129* (50), 15416–15417.

(4) Stöhr, J. *NEXAFS spectroscopy*; Springer Series in Surface Science; Springer: Heidelberg, 1996; Vol. 25.
